# Optical theranostics in ischemic heart disease: from molecular insights to clinical translation

**DOI:** 10.7150/thno.114307

**Published:** 2025-06-09

**Authors:** Yangjie Xiao, Jing Zhang, Lu Sun, Ke Wang, Yan Chen, Qiaojin Zheng, Ying Li

**Affiliations:** 1 Department of Ultrasound, Shengjing Hospital of China Medical University, No. 36 Sanhao Street, Heping District, Shenyang, 110004 Liaoning, China.; 2 Department of Cardiac Surgery, Shengjing Hospital of China Medical University, No. 36 Sanhao Street, Heping District, Shenyang, 110004 Liaoning, China.

**Keywords:** ischemic heart disease, optical coherence tomography, near-infrared fluorescence imaging, photoacoustic imaging, photothermal therapy, photodynamic therapy

## Abstract

Accurate risk stratification of ischemic heart disease (IHD) remains pivotal for mitigating associated global health burdens. Optical theranostics, combining imaging and therapeutic capabilities through light-based technologies, has emerged as a transformative strategy for IHD management. Advanced modalities mainly including optical coherence tomography, near-infrared fluorescence imaging, and photoacoustic imaging enable high-resolution visualization of coronary anatomy, myocardial perfusion, and molecular biomarkers. These modalities complement traditional cardiovascular imaging by providing real-time functional and molecular data with enhanced spatial resolution. Concurrently, phototherapy strategies such as image-guided photodynamic and photothermal therapies show therapeutic potential in preclinical studies. Integrated theranostic platforms now operationalize closed-loop workflows, where diagnostic imaging directly informs therapeutic parameters and monitors treatment response. While challenges persist in clinical translation—including tissue penetration limitations and safety optimization—ongoing developments in contrast agents, device miniaturization, and multimodal integration are accelerating practical applications. This review examines current progress in optical theranostics for IHD, analyzing technical principles, preclinical/clinical implementations, and translational barriers to optimize cardiovascular care through light-based technologies.

## Introduction

Cardiovascular diseases (CVDs), encompassing ischemic heart disease (IHD) and related vascular pathologies, remain the predominant global mortality driver, with IHD alone responsible for approximately 50% of CVD-related fatalities 1. IHD pathogenesis is characterized by coronary atherosclerosis-driven stenosis (CAS), progressing from subclinical plaque formation to myocardial ischemia and infarction 2-4. This pathological continuum underscores the critical need for precision diagnostics that integrate anatomical, functional, and molecular assessments to guide therapeutic interventions.

Current IHD management hinges on dual anatomical-functional evaluation, yet faces critical trade-offs. While invasive coronary angiography remains the gold standard for CAS quantification, its procedural risks and inability to assess hemodynamic significance drive demand for advanced imaging. Computed tomography angiography achieves anatomical precision, yet fails to quantify ischemia. Perfusion imaging modalities such as stress echocardiography, single-photon emission computed tomography (SPECT), magnetic resonance imaging (MRI), and positron emission tomography (PET) address this limitation through pharmacologically-induced hyperemia protocols, enabling myocardial blood flow quantification. However, inherent limitations persist: cumulative radiation exposure, nephrotoxic contrast risks, and temporal/spatial resolution constraints 5. These challenges highlight the unmet need for non-ionizing, multi-scale imaging solutions.

Optical theranostics emerge as a paradigm-shifting approach, integrating light-based diagnostics and therapeutics 6,7. General optical imaging (OI) techniques include optical coherence tomography (OCT), near-infrared fluorescence imaging (NIRFI), photoacoustic imaging (PAI), diffuse optical tomography, bioluminescence imaging, and Cerenkov luminescence imaging. Additionally, biomedical microscopy techniques such as photoacoustic microscopy and fluorescence lifetime imaging microscopy, as well as spectroscopy methods including near-infrared spectroscopy (NIRS) and Raman spectroscopy (RS), also fall within the domain of OI. Among these, OCT, NIRFI, and PAI have gained significant attention due to their promising clinical translatability, making them potential indispensable tools for monitoring IHD. OCT achieves 10-20 μm resolution for vulnerable plaque characterization via intravascular NIR imaging, while PAI quantifies hemoglobin gradients to map myocardial oxygenation. NIRFI enables molecular profiling of inflammatory biomarkers through activatable nanoprobes with picomolar sensitivity 8. Crucially, these modalities overcome traditional limitations through and non-ionizing NIR illumination and real-time intraoperative guidance. Concurrently, phototherapeutic strategies demonstrate therapeutic potential: reactive oxygen species (ROS)-generating photodynamic therapy achieves plaque stabilization in preclinical models, while nanoparticle-mediated photothermal thrombolysis reduces thrombus volume.

Recent reviews have addressed optical theranostics in cardiovascular diseases. Pang *et al.* summarized OI applications for atherosclerosis, myocardial infarction (MI), and valvular pathologies 9. Specialized focus has been placed on intravascular imaging (IVI) for coronary atherosclerosis 10, particularly OCT 11. Additionally, nanotheranostics combining imaging and therapy in CVDs management have been thoroughly reviewed 12,13. Distinctively, our review systematically analyzes both OI techniques and optical therapies across the entire IHD continuum-spanning coronary atherosclerosis, myocardial ischemia, and MI. Through interdisciplinary synthesis of theranostic design principles, molecular insights, and translational evidence from preclinical and clinical studies, we establish optical theranostics as a transformative paradigm for improving IHD diagnosis, targeted treatment, and chronic disease management, while delineating key challenges for future research.

## The pathogenesis and molecular imaging targets of IHD

**Figure 1:**
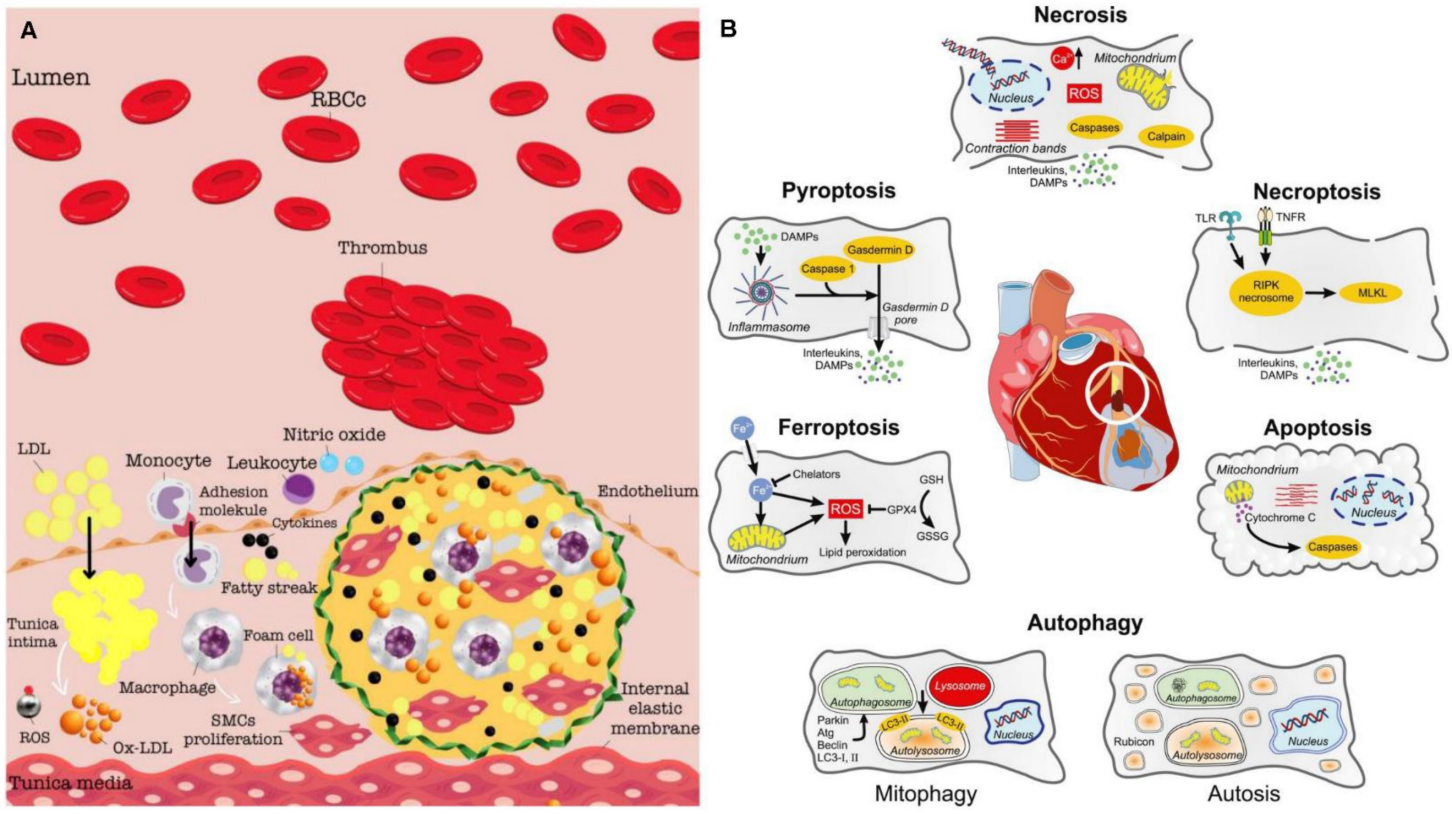
** The pathogenesis and molecular imaging targets of IHD.** (A) The pathogenesis of atherosclerotic plaque and thrombus development. Adapted with permission from 15, copyright 2024 The Author(s). (B) Modes of cardiomyocyte death during myocardial ischemia/reperfusion with their most characteristic features. Adapted with permission from 17, copyright 2024 The Author(s).

The pathobiology of IHD arises from a dynamic interplay of endothelial dysfunction, chronic inflammation, atherosclerotic plaque formation, and ischemic myocardial injury. Central to this process is the progressive impairment of coronary arteries, whose primary role in delivering oxygenated blood to the myocardium is compromised by atherosclerosis—the principal etiological driver of IHD. Atherogenesis initiates with endothelial dysfunction in the arterial intima, marked by diminished nitric oxide (NO) bioavailability due to oxidative stress and reduced endothelial NO synthase activity. This pathophysiological state facilitates subendothelial retention of low-density lipoprotein (LDL) particles, which undergo oxidative modification to form oxidized LDL (ox-LDL). Ox-LDL acts as a potent proinflammatory stimulus, triggering monocyte recruitment via upregulation of adhesion molecules including vascular cell adhesion molecule 1 (VCAM-1), intercellular adhesion molecule 1 (ICAM-1) and P-/E-selectins. Engulfment of ox-LDL by infiltrating macrophages via scavenger receptors generates lipid-laden foam cells, forming early fatty streaks that constitute the nascent atherosclerotic lesion **14**. Proinflammatory cytokines drive phenotypic modulation of vascular smooth muscle cells (VSMCs), prompting their migration from the media to the intima. These VSMCs proliferate and secrete extracellular matrix (ECM) components, forming a fibrous cap overlying a necrotic lipid core—a hallmark of mature atheroma. Concurrently, matrix metalloproteinases (MMPs) degrade collagen and elastin within the fibrous cap, destabilizing plaque architecture. Plaques that grow slowly tend to stabilize and develop thick fibrous caps, reducing their likelihood of rupture. However, plaques with thin fibrous caps are more prone to rupture, which exposes thrombogenic material that activates platelets and the clotting cascade. This can cause acute thrombosis and rapid narrowing of the arterial lumen **15** (Figure 1A). The progressive narrowing of coronary arteries, due to the development of both stable and unstable plaques, substantiates the clinical manifestations of IHD, including angina, acute coronary syndromes, and MI **16,17** (Figure 1B). Emerging molecular imaging modalities now enable visualization of key pathobiological processes from atherosclerotic plaque formation to myocardial MI **18,19**. Convergence of these diagnostic innovations with mechanism-targeted therapies heralds a paradigm shift in decoding IHD pathogenesis and advancing precision-guided interventions.

Super-resolution optical microscopy, a transformative imaging modality that overcomes the diffraction barrier, enables nanoscale visualization of myocardial cell structures and pathological mechanisms in myocardial ischemia. Pioneering techniques such as ​Stimulated Emission Depletion Microscopy (STED)​​ for real-time organelle dynamics, ​Photoactivated Localization Microscopy (PALM)​​ for genetically encoded protein tracking, and ​Stochastic Optical Reconstruction Microscopy (STORM)​​ for multicolor molecular mapping have revolutionized cardiac research by resolving subcellular alterations previously inaccessible to conventional imaging. STED enabled live mapping of transverse tubule membrane structures in heart failure post-MI 20 and precise lysosome identification in myocardium during myocardial ischemia/reperfusion injury (MI/RI) 21. PALM facilitated semi-quantitative analysis of the α-actinin network in human induced pluripotent stem cell-derived cardiomyocytes 22. STORM revealed nanoscale organization of ion channels critical for excitation-contraction coupling, including L-type calcium channels, ryanodine receptor type 2, and junctophilin-2 23, as well as cluster size and density of cardiac sodium channel Nav1.5 24.

## OCT for IHD detection

### Principle and equipment of OCT

IVI technologies transcend conventional angiography by enabling three-dimensional coronary assessment, integrating luminal geometry, plaque composition, and vascular wall dynamics 15,16. These tomographic modalities overcome the planar limitations of luminography through plaque characterization, procedural guidance, and risk stratification. While intravascular ultrasound (IVUS) provides real-time cross-sectional imaging for plaque burden quantification and percutaneous coronary intervention (PCI) optimization 25. OCT, as another emerging IVI technology, delivers superior resolution for microstructural analysis, enabling precise identification of vulnerable plaque features and stent apposition quality 11.

**Figure 2:**
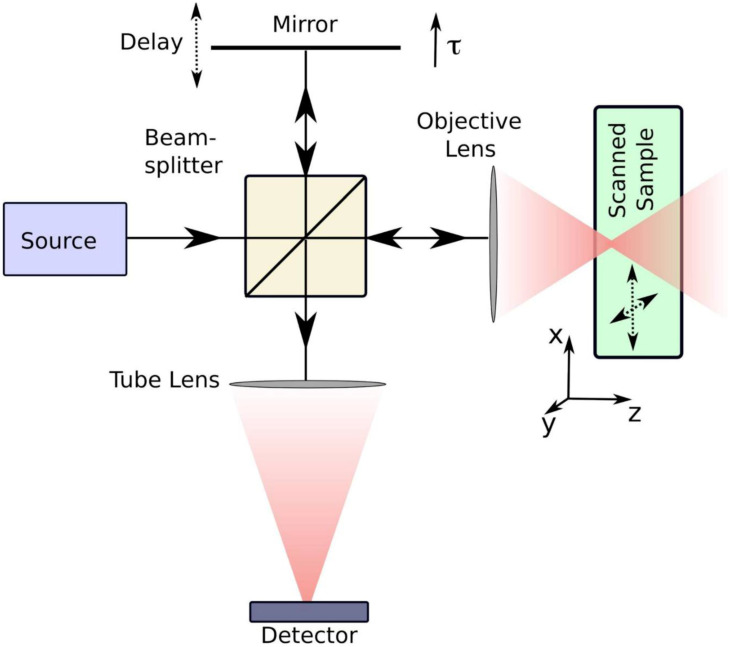
** A basic illustration of an OCT system.** Light traveling in one arm of a Michelson interferometer is focused into the sample. The length of the reference arm can be adjusted using a moveable mirror. The reference light and the light backscattered from the sample interfere at the detector. Adapted with permission from 27, copyright 2008 The Author(s).

OCT operates on interferometric principles, utilizing NIR light to detect depth-resolved tissue backscattering. The system splits light into reference and sample arms, with returning photons generating interference patterns when optical path differences fall within the coherence length. Constructive interference amplifies signals from specific tissue depths, while destructive interference suppresses background noise. Radial scanning through a rotating intracoronary probe reconstructs cross-sectional vascular architecture with micron-level precision **26,27** (Figure 2). OCT systems are primarily categorized into two types: Time Domain OCT (TD-OCT) and Frequency Domain OCT (FD-OCT). TD-OCT uses a broadband light source, and the reference mirror is mechanically moved to create varying time delays. This process is relatively slow because the system captures light reflection at one depth point at a time. FD-OCT employs a wavelength-swept laser as the light source, with the fixed reference mirror. This technological advancement provides an enhanced signal-to-noise ratio and faster sweeps, resulting in significantly improved image acquisition and catheter withdrawal speeds as compared to those of TD-OCT. Among the current IVI modalities, OCT offers the highest resolution (axial 10-20 μm and lateral 20-90 μm) at the expense of low penetration depth (1-2 mm) **28**.

### Application of OCT in coronary atherosclerosis

**Figure 3:**
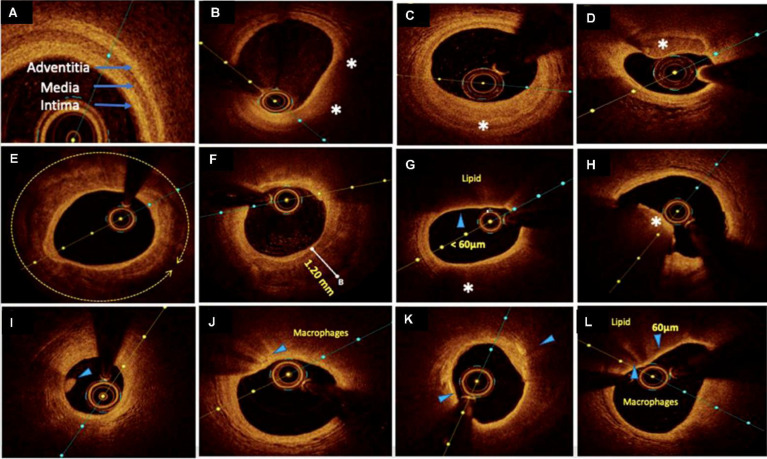
** Representative OCT images with various plaque morphologies.** (A) Normal coronary, (B) lipid-rich plaque, (C) fibrotic plaque, (D) calcific nodule, (E) near 360° arc of calcific plaque, (F) deep calcium deposition, (G) thin cap fibroatheroma (TCFA), (H) intraluminal red thrombus, (I) intraluminal white thrombus, (J) bright spots or bands at the boundary between the fibrous cap and lipid core suggestive of macrophages, (K) bright signal-rich cholesterol crystals, and (L) vulnerable plaque formed by a large lipid pool covered by TCFA with macrophage infiltration. Areas of interest were highlighted by arrows and asterisks. Adapted with permission from 29, copyright 2022 The Author(s).

OCT imaging of coronary atherosclerosis reveals tissue-specific optical attenuation profiles that govern visualization efficacy. High-attenuation tissues such as lipid-rich plaques restrict light penetration due to strong scattering and absorption, whereas collagenous and calcified components permit deeper NIR light propagation. This variable penetration capacity (0.1-2.0 mm) enables OCT to delineate plaque architecture through detailed cross-sectional visualization of tissue composition and stratified morphology 21 (Figure 3).

In IHD management, OCT's micrometer-scale resolution provides critical insights across the clinical continuum. During acute coronary syndrome evaluation, it identifies culprit lesion pathology—including TCFA (<65 μm), plaque rupture, and thrombus formation—distinguishing erythrocyte-rich (red thrombi, high-attenuation) from platelet-dominant thrombi (white thrombi, signal-rich). Thrombi are visualized as irregular clusters suspended in the lumen or adhered to the vessel wall, critical for assessing lesion stability. This capability directly informs therapeutic strategies by correlating morphological features with pathophysiological mechanisms 11,30,31. For PCI, OCT serves as a comprehensive guidance platform. Pre-procedural imaging characterizes calcification patterns (thickness, arc, length), guiding lesion preparation via rotational atherectomy or lithotripsy to optimize calcium fracture. Intraprocedural monitoring ensures optimal stent deployment through real-time assessment of expansion uniformity and wall apposition, mitigating risks of under-expansion and malapposition. Post-intervention analysis verifies procedural endpoints by detecting residual dissection, geographic miss, or stent strut malposition—key determinants of restenosis and stent-related thrombosis 28,32. OCT's value escalates in complex scenarios: bifurcation lesions benefit from branch ostium visualization, while overlapping stents require precise strut alignment verification. Through layered plaque characterization and device-tissue interaction analysis, OCT transforms interventional cardiology from anatomical correction to precision-guided vascular reconstruction 33.

As a clinically established IVI technology, numerous high-quality clinical trials have demonstrated the value of OCT in evaluating coronary plaque characteristics and guiding interventional therapy, providing robust evidence-based guidance for standardized clinical implementation. The CLIMA Study​ defined high-risk plaque features using OCT and identified four OCT criteria (minimum lumen area, fibrous cap thickness, lipid arc and macrophage infiltration) as predictors of future cardiovascular events 34. ​EROSION III Study​ assessed OCT-guided stent necessity in non-obstructive ST-segment elevation MI and found OCT reduced unnecessary stent implantation by ​15%​​ and improved residual stenosis management 35. OCTOBER trial​ evaluated OCT-guided PCI for complex bifurcation lesions, and demonstrated OCT reduced 2-year major adverse cardiovascular events and target lesion revascularization, supporting its routine use in bifurcation interventions 36. The ​ILUMIEN series​ is a collection of landmark clinical trials investigating the utility of OCT​​ in optimizing PCI, particularly compared to traditional angiography and IVUS 37-41.

**Figure 4:**
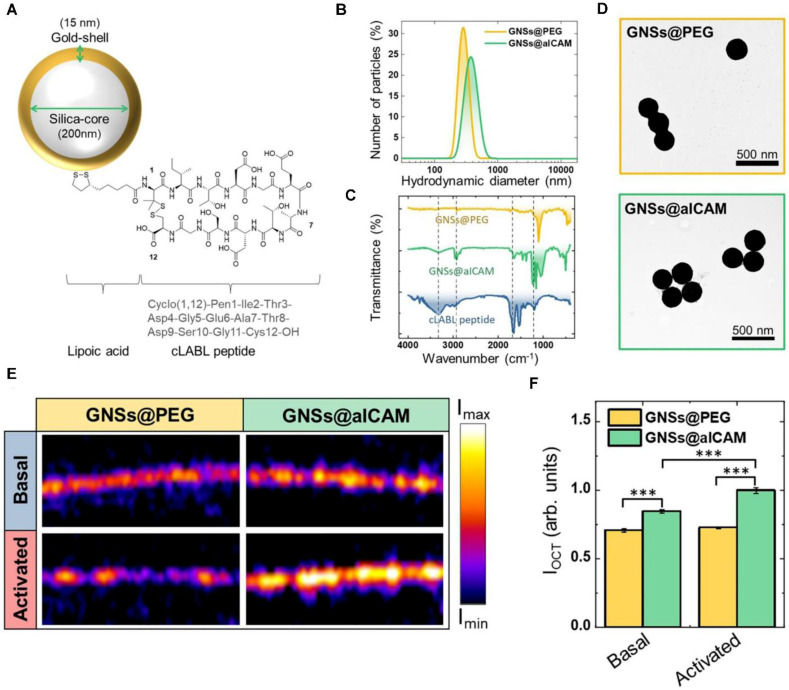
** Optical detection of atherosclerosis at molecular level by OCT.** (A) GNSs@aICAM schematic drawing.(B)Hydrodynamic diameter of GNSs@PEG and GNSs@aICAM. (C) Fourier transform infrared spectra of GNSs@PEG, GNSs@aICAM, and cLABL peptide. (D) Transmission electron microscopy (TEM) images of GNSs@PEG (top) and GNSs@aICAM (bottom). (E) OCT images of basal and activated HMEC cells monolayers incubated with GNSs@PEG or GNSs@aICAM. (F) OCT total intensity of the cell cultures areas for both basal and activated cells. Adapted with permission from 42, copyright 2022 The Author(s).

The evolution of OCT now extends to molecular-level interrogation through targeted contrast agents that bind atherosclerosis-specific biomarkers. The seminal work by Muñoz-Ortiz *et al.* utilizes ICAM-1-targeted gold nanoshells, exploiting vascular inflammation-driven receptor overexpression. This molecular OCT strategy enables spatially resolved visualization of early inflammatory cascades, bridging histological insight with IVI—a critical advancement for preemptive risk stratification and personalized therapeutic planning in coronary atherosclerosis 42 (Figure 4). Building upon its established role in high-resolution plaque characterization and stent optimization, OCT is redefining diagnostic paradigms in IHD. The integration of molecular imaging capabilities positions OCT as a multimodal platform, transitioning from structural assessment to pathophysiology-driven precision cardiology. Future developments aim to synergize nanoscale biomarker detection with real-time intervention guidance, ultimately enabling targeted anti-inflammatory therapies and dynamic monitoring of treatment response.

OCT revolutionizes IHD management by superior resolution for microstructural analysis, enabling precise identification of vulnerable plaques and stent apposition, improving PCI guidance and outcomes in complex lesions. Clinical trials demonstrate its ability to reduce stent-related complications and predict cardiovascular events. Emerging molecular OCT enhances early detection of biomarkers, advancing personalized risk stratification and therapy. However, its shallow penetration depth limits visualization in large vessels or deep plaque structures, particularly in lipid-rich plaques. Technical complexity and reliance on NIR light restrict its use in in complex scenarios. Future advancements in ​artificial intelligence (AI) integration and ​hybrid imaging systems​ promise to address current gaps, solidifying OCT's role in precision cardiology.

## NIRFI for IHD detection

### Principle and equipment of NIRFI

NIRFI is an advanced optical molecular imaging technique that employs photon-induced electronic transitions in fluorophores for molecular-level tissue interrogation. Operating within 650-1000 nm wavelengths, this modality detects endogenous NIR autofluorescence (NIRAF) from biological chromophores or utilizes exogenous agents targeting biomarkers. The technique capitalizes on Stokes shift principles, where emitted photons exhibit longer wavelengths than excitation light, enabling spectral separation via emission filters and high-sensitivity charge-coupled device (CCD) detection. Clinical translation benefits from NIRAF's inherent safety profile, enabling atherosclerotic plaque identification through natural fluorophore signatures. Exogenous contrast agents include NIR fluorophores alone or conjugated to antibodies, peptides, or small molecules that target specific biomarkers. Examples of such contrast agents are indocyanine green (ICG) and fluorophore-labeled ligands targeting molecular markers such as ICAM-1, VCAM-1, or integrin. NIRFI enables real-time visualization of molecular and tissue features, which negates the need for time-consuming image reconstruction or post-processing steps.

Planar imaging systems are fundamental tools for two-dimensional (2D) fluorescence imaging and are recognized for their simplicity, real-time imaging capability, and versatility in detecting specific fluorophores. One or more light sources are used to excite fluorophores within the target tissue. The emitted fluorescence is captured by a CCD camera, which generates a 2D fluorescence image in real time. The primary drawback of planar imaging is the inability to determine the depth of fluorescence signal origin; it is also less suitable for the precise quantification of fluorophore distribution, particularly in thicker or more complex tissues 43. *In vivo* NIRFI is particularly an attractive approach for human coronary arterial molecular imaging. The 2D intravascular NIRFI strategy using a rotational and automated pullback NIRF intravascular catheter device can facilitate nanomolar-sensitive, intra-arterial molecular imaging in vessels with a larger diameter, thus providing high-resolution *in vivo* spatial mapping of coronary-sized arteries 44,45.

**Figure 5:**
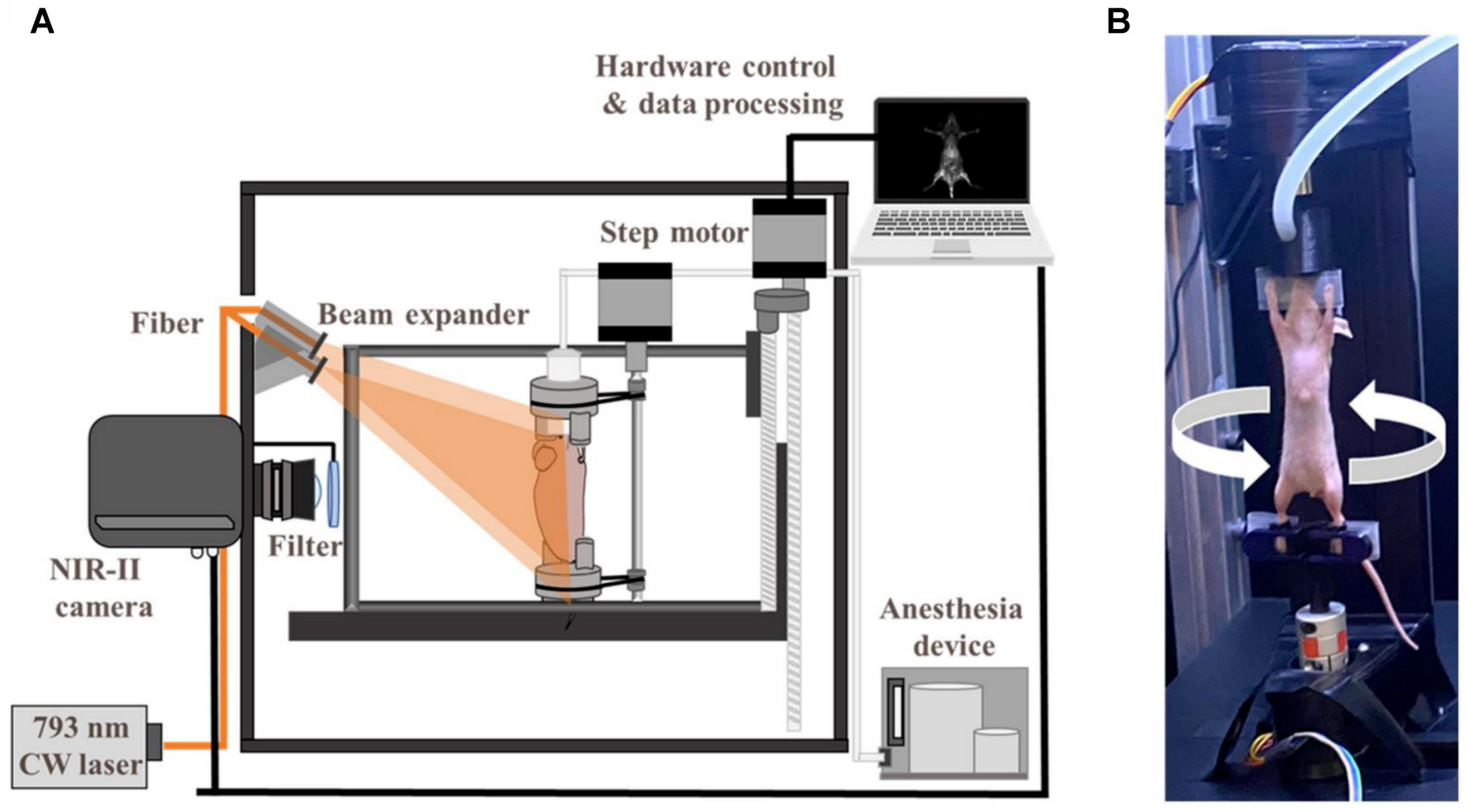
** Schematic diagram of the 3D NIRFI system.** (A) Schematic diagram of Home-built 3D NIR-II fluorescence rotational stereo imaging system. (B) Photograph of the experimental setup of *in vivo* optical imaging. Adapted with permission from 48, copyright 2023 The Author(s).

The emergence of second NIR window (NIR-II, 1000-1700 nm) fluorescence imaging has overcome fundamental limitations of conventional NIR-I techniques through optimized photon-tissue interactions. NIR-II's unique optical profile—characterized by enhanced tissue penetration, suppressed autofluorescence, and minimized scattering—enables superior *in vivo* imaging fidelity **43,46**. Stereoscopic NIR-II systems achieve three-dimensional (3D) vascular mapping through dual-perspective InGaAs camera arrays and precision motion control, reconstructing depth-resolved anatomical architectures **47,48** (Figure 5). Complementary confocal configurations further enhance resolution for microvascular interrogation, as demonstrated in cerebral circulation studies **49**. These advancements position NIRFI as a transformative platform for intraoperative molecular guidance and therapeutic monitoring in cardiovascular interventions.

### Intravascular NIRFI of the coronary artery

**Figure 6:**
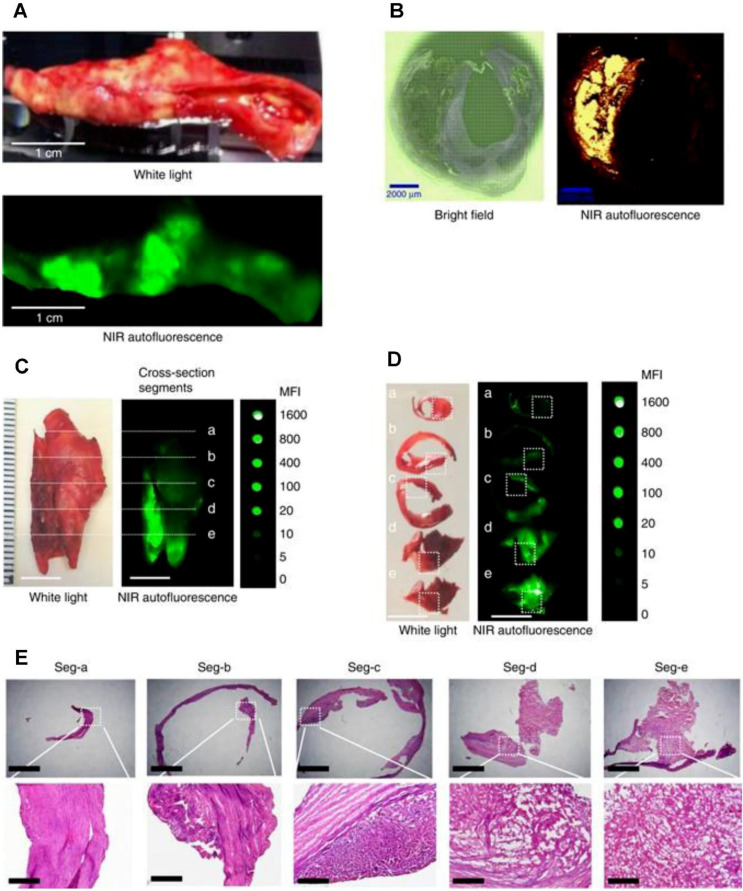
** NIRAF imaging of human carotid endarterectomy samples and representative histology in five cross section segments.** (A) Macroscopic sample of a fresh human carotid endarterectomy (CEA) specimen under white light (upper photo) and fluorescence imaging (lower image) showing localized areas of NIRAF. (B) Paraffin section of human CEA specimen: Bright field (left) and areas of NIRAF (right). (C) Fresh human CEA specimen under white light (left) and its NIRAF image (middle) shown in comparison with a serial dilution of IRDye800CW (right). The same sample was sectioned into five segments. (D) These five plaque segments under white light (left) and their corresponding NIRAF images (middle) were again shown in comparison with the IRDye800CW (right). (E) Histology of these five plaque segments with increasingly advanced pathology stained with hematoxylin and eosin. Adapted with permission from 50, copyright 2017 The Author(s).

Intravascular NIRFI has emerged as a transformative modality for molecular-level characterization of atherosclerotic plaques, resolving pathophysiological features including inflammatory activation, oxidative stress dynamics, and endothelial barrier dysfunction **44**. While the precise biomolecular origins of NIRAF remain under investigation, clinical studies validate its correlation with high-risk plaque phenotypes. Elevated NIRAF signals associate with necrotic core presence and intraplaque hemorrhage—histopathological hallmarks of plaque vulnerability—while ox-LDL-driven oxidative stress further amplifies fluorescence signatures **50,51** (as shown in Figure 6). In clinical research, intracoronary NIRAF is usually combined with OCT. In the first-in-human study with intracoronary NIRAF-OCT, Ughi *et al.* demonstrated that dual-modality NIRAF-OCT imaging is a robust tool for the *in vivo* assessment of coronary artery disease. The ability to detect a unique human coronary autofluorescence signature and integrate it with structural imaging substantially enhances the understanding of atherosclerosis **52**. This dual-modality strategy has been extended to therapeutic monitoring, exemplified by peroxisome proliferator-activated receptor (PPAR)-α agonist pemafibrate reducing stent-induced protease activity—a key mediator of restenosis—validating NIRAF-OCT's utility in evaluating anti-inflammatory interventions **53**.

Recent advances in exogenous NIR fluorophore development, encompassing small molecule dyes and engineered nanoparticles, have significantly advanced NIRFI for atherosclerotic plaque and thrombus characterization. Established molecular targets for this modality include macrophage infiltration, cathepsin protease activity, ox-LDL, and endothelial barrier dysfunction 45. Preclinical validation in large-animal models with coronary artery dimensions analogous to humans has addressed critical limitations in translational applicability 54. The integration of ICG, the first FDA-approved NIR dye, with intracoronary NIRFI-OCT by Verjans *et al.* enabled real-time visualization of endothelial integrity loss, fibrous cap disruption, and neovascularization 55. This platform further demonstrated efficacy in quantifying plaque inflammation and drug-eluting stent-induced vascular responses in dynamic coronary environments, underscoring its clinical potential for vulnerable plaque assessment 56. Concurrently, targeted molecular probes continue to diversify. Hemadou *et al.* engineered an scFv-Fc-2c/Alexa Fluor 647 probe for macrophage-associated inflammation imaging 57. while Bertrand *et al.* developed targeted probes using anti-ICAM-1 single-domain antibody (SDA)/collagen-binding hairpin peptide (CBHP) to synergistically evaluate plaque composition and inflammatory activity via NIRFI- IVUS 58. Parallel innovations by Khamis *et al.* yielded ox-LDL-targeted probes LO1-750 and LO9-750, permitting quantitative assessment of oxidative stress 59,60. Fibrin-targeted peptide (FTP11) conjugated to a NIRF dyes were designed and synthesized to validate *in vitro* through noninvasive NIRFI, demonstrating that drug-eluting stents exhibited greater fibrin deposition 61,62.

Nanoparticle engineering has progressed in developing exogenous NIR fluorophore. Stein-Merlob *et al.* engineered ultrasmall superparamagnetic iron oxide nanoparticles (CLIO) conjugated with CyAm7 fluorophores, enabling intravascular NIRFI detection of high-risk plaques in human-scale coronary atheroma models 63. Concurrently, Hu *et al.* developed semiconductor quantum dots (IR-QDs) (1.55-1.87 μm emission) that synergize NIRFI-OCT imaging through single-wavelength excitation (1.3 μm), achieving concurrent backscattering analysis and high-resolution luminescence mapping 64. A notable therapeutic advancement involves PPAR-γ-targeted NIRFI-OCT nanodrugs that demonstrate dual functionality in stabilizing inflamed atherosclerotic plaques while providing quantitative metrics for treatment monitoring 65. Further developments in molecular targeting include Zou *et al.*'s biomimetic Fe_3_O_4_-Cy7 nanoparticles engineered with macrophage membrane coatings, which exhibit CCR2-mediated specificity for atherosclerotic plaque visualization through NIR-MRI multimodal imaging 66.

The identification of intracoronary thrombus and atherothrombosis is central to the diagnosis of acute MI 67. Therefore, biomarkers of platelet activation, fibrin generation and even coagulation factors have attracted major attention as molecular targets for molecular imaging of arterial and venous thrombosis 68. The FXIIa-targeted NIR probe 3F7-NIR enables specific arterial/venous thrombus detection *in vivo/ex vivo* with significantly enhanced imaging signals, advancing early diagnosis and guiding antithrombotic therapy with NIR imaging 69,70. Lim *et al.* developed a recombinant Targ-Cy7 fluoroprobe (scFvTarg-Cy7) enabling NIR imaging of thrombi with 8.2-fold higher specificity, facilitating longitudinal efficacy monitoring and pulmonary embolism detection via an optical platform for preclinical thrombosis research 71. Research focusing on coronary artery-specific modeling remains limited. Bai *et al.* advanced thrombus detection methodologies through PLGA-cRGD-PFH-ICG nanoparticles that demonstrate activatable bimodal NIRFI-intravascular PA (IVPA) imaging upon low-intensity focused ultrasound stimulation, establishing a novel framework for microthrombus identification in clinical risk stratification 72. These nanoplatform innovations, when integrated with next-generation imaging platforms including NIRFI-OCT, NIRFI-IVUS, and dual NIRFI-MRI systems, represents a significant progress in atherosclerosis imaging (Table 1).

### NIRF coronary artery angiography

Beyond NIRFI for coronary atherosclerosis, ICG application has been shown to be a feasible approach to assess blood flow in coronary vessels and bypass grafts and to analyze myocardial perfusion 73. Fluorescence coronary angiography represents a highly sensitive and reproducible approach and a valuable technique for intraoperative quality assessment in coronary artery bypass grafting 74,75. ICG NIRF complex angiography and perfusion analysis enables to identify the characteristics of stenosis in the target vessel through visualization of the physiological response to grafting 76. A quantitative NIRF assessment with a high-resolution NIRF device can enhance the detection of stenosis at the anastomotic site, and high-resolution NIRFI was found to be effective in identifying arterial dissection in grafts 77. Methylene blue (MB), another FDA-approved fluorophore, has been shown to be an optimal NIR fluorophore for the direct visualization of coronary arteriography and cardiac perfusion. It also facilitates critical functional assessments during cardiac surgery 78.

### NIRFI for the myocardium

**Figure 7:**
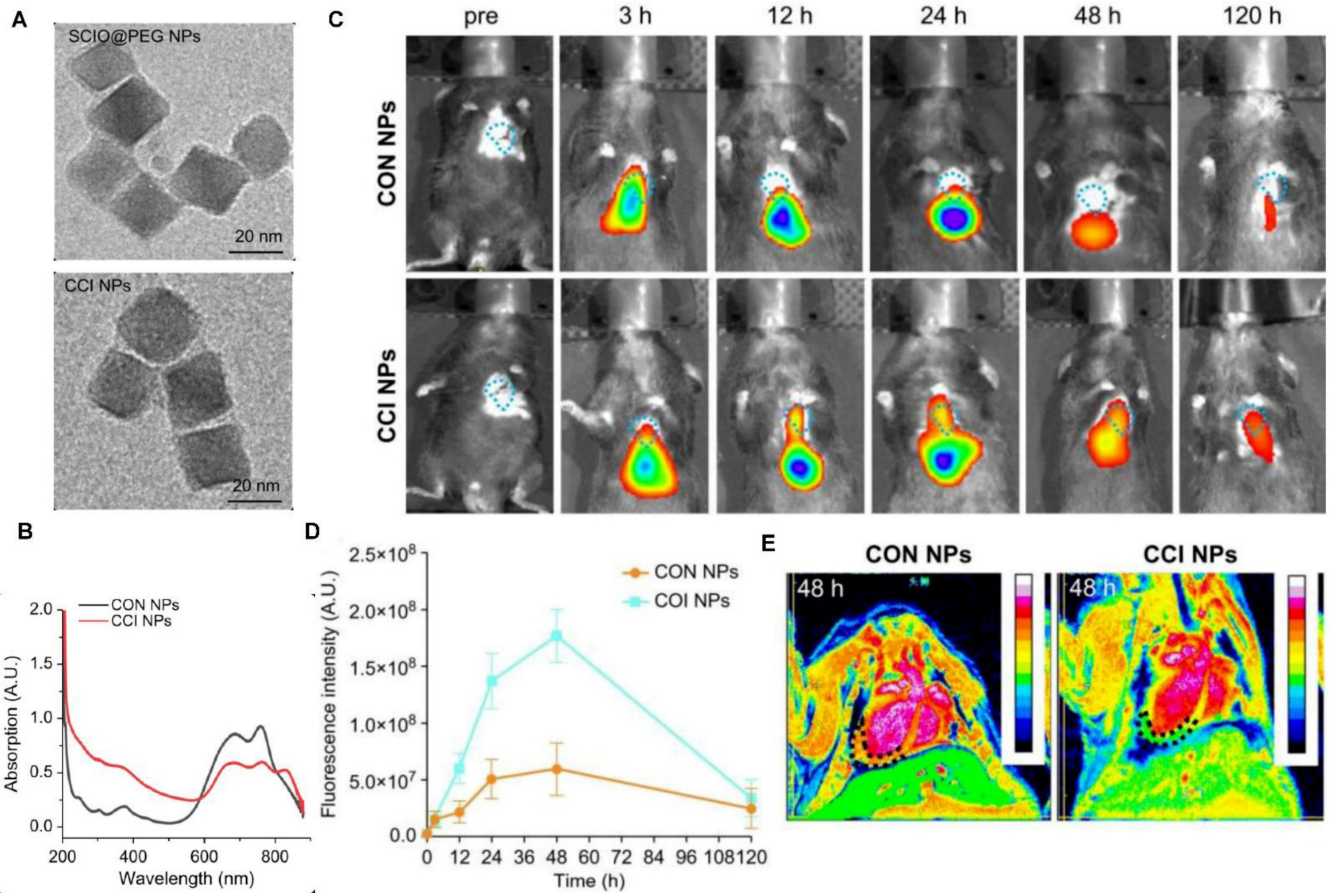
***In vivo* NIRFI/MRI multimodal imaging of the MI/RI mouse model.** (A) TEM images of SCIO@PEG NPs and CCI NPs. (B) The UV-Vis absorbant spectra of CON NPs and CCI NPs. (C) NIRF images of the MI/RI mouse model at different time points *in vivo* from different groups (CON NPs vs. CCI NPs). (D) Quantitative comparison of fluorescence intensities of CON/CCI NPs. (E) Images of the MI/RI mouse model injected CON/CCI NPs obtained from MRI at 48 h post-injection. The location of the cardiac tissue with a locally observable low intensity is circled by black dots. Adapted with permission from 85, copyright 2024 The Author(s).

NIRF has also been used for the imaging of the myocardium, including direct myocardial perfusion for blood flow, determination of myocardial ischemia induced cell injury, and detection of changes in the myocardial microenvironment. Normal myocardial cells have robust mitochondria, and lipophilic cations are taken up passively by the myocardium tissue. F16 and its derivatives are novel small molecule dyes, which function as mitochondria-targeting lipophilic cations and can be used for myocardial perfusion imaging to evaluate myocardial blood supply and cell viability **79**. Myocardial cell-related biomarkers, including biomarkers for apoptotic and necrotic cells, nucleic acids, autophagosomes, and ferroptosis, can be visualized by NIRFI. Wang *et al.* engineered an aggregation-induced emission probe (TPABTBP) to monitor lipid droplet (LD) dynamics during MI/RI-induced ferroptosis, revealing LD accumulation peaks at early myocardial reperfusion (0-9h) but declines via lipophagy in late stages (> 24h), with LD breakdown inhibition significantly suppressing cardiomyocyte lipid peroxidation **80**. Acharya *et al.* engineered the ApoPep-1-FITC/Flamma 774 probe, which detects cell death signatures by targeting histone H1 exposed on apoptotic and necrotic cells during acute MI onset, providing early-phase NIRF signal quantification **81**. Tian *et al.* demonstrated that *in vivo* Cy5.5-annexin V fluorescence imaging effectively detects MI/RI-induced apoptosis in mice, validating its utility as a noninvasive method for cardiac apoptosis assessment **82**. The dextran-conjugated thiazole orange (Dextran-TO) nanoprobe demonstrates dual-functionality through nanomolar-affinity nucleic acid scavenging and anti-inflammatory cytokine modulation in macrophages, enabling precise infarct localization via NIRFI **83**. Chen *et al.* developed an iron oxide-core dual-modal NIRFI/MRI platform integrating cathepsin-cleavable arginine-rich peptides with Cy5.5 fluorophores, permitting injury-specific quantification of autophagic flux through combined NIRFI/MRI detection **84**. SCIO-ICG-CRT-CPP nanoparticles showcased MI/RI-specific targeting coupled with a magnetic particle imaging (MPI)-MRI-NIRFI multimodal imaging triad, creating a transformative platform for dynamic ferroptosis assessment in MI/RI **85** (Figure 7).

**Figure 8:**
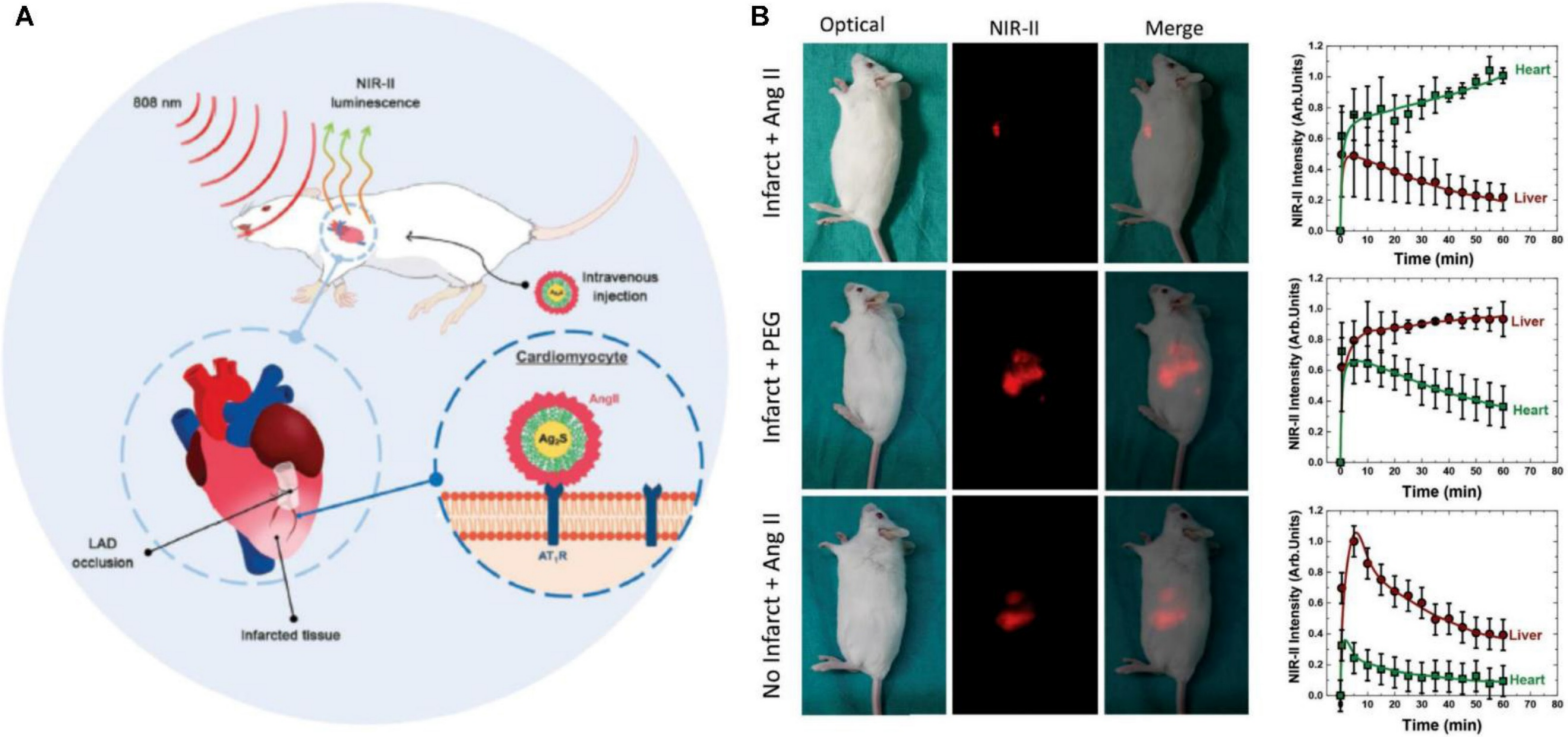
** Imaging of Acute MI by NIR-II luminescent nanodots** (A) Schematic representation of the experimental procedure followed in this work for *in vivo* imaging of acute infarct. (B) Optical, NIR-II fluorescence and merged images of representative mice (acute infarct, without any infarct) followed by an intravenous injection of AngII-functionalized Ag_2_S NDs or PEGylated Ag_2_S NDs corresponding to the three scenarios were presented. Corresponding time course of the average NIR-II luminescence intensity generated at the heart and liver. Adapted with permission from 92, copyright 2020 WILEY-VCH Verlag GmbH & Co. KGaA.

NIRFI with conditionally active probes is an efficient approach to visualize and monitor microenvironmental changes in the myocardium after ischemia or infarction. Notably, ICG accumulates in the areas of MI/RI due to an increase in vascular permeability and extravasation of the dye; this feature is used for the *in vivo* visualization of MI 86. Building on this vascular targeting paradigm, Chen *et al.* engineered an MMP2/9-activatable NIRF probe that maps MMP activity dynamics in post-MI myocardium, providing spatiotemporal resolution of protease activity through its protease-cleavable molecular design 87. Concurrently addressing oxidative stress, Lu *et al.* engineered the BBEB probe, a peroxynitrite (ONOO⁻)-responsive NIRFI system that demarcates acute MI lesion boundaries with nanomolar sensitivity, enabling precise visualization of early-stage injury for antioxidant therapy development 88. Complementarily, the NOF5/Cy3-SCLMs probe achieves real-time ONOO⁻ monitoring during reperfusion through passive infarct targeting, resolving oxidative stress dynamics with second-scale temporal resolution 89. Ziegler *et al.* developed self-assembled ROS responsive fluorescent nanoparticles composed of chlorin e6, luminol, and polyethylene glycol, which exhibited targeted accumulation in ischemic myocardium within 24 hours post-reperfusion in a murine coronary ligation model, highlighting potential localized MI/RI therapeutic efficacy while minimizing systemic toxicity 90. Expanding into hypoxic microenvironment imaging, Fan *et al.* engineered Pep/BDP-NO₂@Lip, a liposomal nanoprobe incorporating a cardiac-targeting peptide and nitrobenzene-BODIPY fluorophore, specifically designed for dual hypoxia-upregulated AngII receptor targeting and NIRFI in ischemic myocardium 91. Further advancing this approach, bioengineered AngII-conjugated Ag₂S nanodots leverage NIR-II imaging's rapid acquisition to achieve submillimeter-scale spatial resolution in acute MI models, enabling precise ischemic tissue delineation 92 (Figure 8).

For multimodal integration, MnO-based dual-modal nanoparticles labeled with Cy5.5 serve as a highly promising MRI contrast agent for the precise and accurate detection of infarcted regions in the myocardium. NIRFI showed that the MnO nanoparticles exhibit preferential accumulation in the infarcted myocardium 93. Song *et al.* engineered a fibrin-targeted multimodal nanoagent (CREKA-conjugated SPIO-fluorophore) enabling dual MRI/NIRFI of microthrombosis in MI/RI models, demonstrating selective accumulation in fibrin-rich microthrombi and potential for both molecular imaging of microcirculatory disorders and targeted fibrinolytic therapy 94. Simultaneously, the magnetofluorescent nanoparticle CLIO-Cy5.5 can be taken up by macrophages that infiltrate the infarcted myocardium; the absorbed nanoparticles can then also be imaged by NIRF and MRI bimodal imaging, thus expanding the role of fluorescence imaging in the heart 95. In the domain of coronary microvascular dysfunction (CMD), ICG-encapsulated fibrin-targeted microbubbles (T-MBs-ICG) achieve dual-modal NIRF/ultrasound imaging through surface-conjugated fibrin-specific peptides, demonstrating selective CMD biomarker detection *in vitro* 96. Complementing this strategy, ischemic myocardium-targeting peptide (IMTP)-guided ICG nanobubbles (IMTP/ICG NBs) enable tri-modal fluorescence/ultrasonic/photoacoustic ischemia localization in CMD models, achieving high spatial resolution 97. Concurrently, Zhao *et al.* engineered osteopontin-targeted phase-change nanoparticles (OPN@PFP-DiR NPs) that synergize ultrasound cavitation with NIRF emission to resolve fibrotic myocardial remodeling through OPN biomarker colocalization 98. Collectively, NIRFI, combined with other modalities such as MRI or ultrasound, has shown superior capability to detect myocardial ischemia and MI with high specificity and sensitivity. The probes leverage pathological changes such as vascular permeability, hypoxia, and macrophage infiltration or use molecular markers such as ONOO⁻ and MMPs to enhance imaging accuracy.

Cell therapy for restoring lost cells has been proposed as a treatment for CVDs, particularly IHD, which cause a substantial loss of cells. Because of the low cell retention and survival rates associated with direct injection, cell therapy using biomaterials as cell carriers has received increasing attention for its enhancement of cell delivery and retention at targeted sites. NIRFI for tracking the behavior of transplanted cells *in vivo* is also a feasible approach 99. Li *et al.* demonstrated that DiD-labeled mesenchymal stem cells (MSCs) improved myocardial retention and cardiac function in rat MI models, with NIRFI validating prolonged engraftment 100. In multipotent progenitor cells (MPCs) therapy, IR-786-based NIRFI revealed intracoronary delivery kinetics dependent on cellular subpopulations, directly correlating with therapeutic efficacy 101. Furthermore, cardiac progenitor cell-derived extracellular vesicles (CPC-EVs) labeled with Alexa Fluor 790/647 exhibited stable myocardial retention post-intramyocardial injection, as confirmed by real-time NIRFI monitoring 102.

For therapeutic cargo tracking, Lu *et al.* engineered photoluminescent mesoporous silicon nanoparticles (PMSNs-siRNA-PEI) for simultaneous CCR2 silencing and NIRFI-guided MSC tracking, demonstrating synergistic anti-inflammatory effects post-AMI 103. Dil-labeled CD47-engineered MSC-EVs (CD47-EVs) loaded with miR-21a via electroporation demonstrated prolonged circulation, enhanced cardiac targeting, and therapeutic efficacy by suppressing apoptosis, reducing inflammation, and improving cardiac function, establishing a novel two-step EV delivery platform for MI/RI, visulized by NIRFI 104. Concurrently, Wang *et al.* demonstrated that angiotensin 1 peptide-conjugated CdSe/ZnS quantum dots effectively deliver cystathionine-γ-lyase plasmids to the myocardium, enhancing localized hydrogen sulfide production to reduce MI/RI by suppressing endoplasmic reticulum stress and mitophagy, offering a targeted, side effect-free therapeutic strategy for cardiac protection 105. The theranostic HI@PSeP-IMTP nanoprobe co-delivering hesperetin/ICG enhances NIR/PA signals in MI/RI regions while suppressing oxidative stress and key inflammation markers via diselenide bonds/hesperetin synergy, demonstrating integrated diagnostic-therapeutic efficacy against MI/RI 106. Sun *et al.* developed IMTP/SS31-conjugated Au-Se core-shell nanostructures (AS-I/S NCs) that integrate mitochondrial antioxidant delivery with dual-modal NIRFI/PAI tracking, effectively mitigating MI/RI 107. NIRFI is a versatile and robust tool for studying myocardial perfusion, and ischemic injury. The ability of NIRFI to utilize a wide variety of fluorophores (small molecules, peptides, and nanoprobes) makes it suitable for targeting specific markers of injury or disease. When combined with other imaging modalities, NIRFI provides a comprehensive view of cardiac function and pathology in IHD, while its applications in cell therapy and drug delivery tracking further extend its clinical relevance 92 (Table 2).

NIRFI enables real-time, high-sensitivity visualization of molecular and tissue features without complex post-processing, leveraging endogenous autofluorescence or exogenous agents targeting biomarkers. Its integration with modalities enhances diagnostic accuracy for atherosclerotic plaques, thrombus, and myocardial injury. The technique also supports therapeutic monitoring, cell therapy tracking, and multimodal imaging, offering versatility with diverse fluorophores and nanoprobes. However, its clinical adoption requires addressing penetration limits, probe optimization, and cost barriers. Planar NIRFI systems lack depth resolution and struggle with precise fluorophore quantification in thick or complex tissues. While exogenous agents, though target-specific, face challenges such as biocompatibility, toxicity, and regulatory hurdles. Clinical translation of advanced NIR-II systems may be limited by cost and procedural complexity, and many applications remain preclinical, requiring further validation for widespread use. Future advancements in NIR-II fluorophores, advanced probe engineering, AI-driven multimodal integration, and standardized protocols will bridge translational gaps.

## PAI for IHD detection

### Principle and equipment for PAI

PAI is a type of OI that utilizes the photoacoustic effect to transform light energy into acoustic waves. A pulsed laser beam illuminates the target tissue, and optical chromophores (such as hemoglobin, melanin, or other molecules in the tissue) absorb light energy. The absorbed light energy is rapidly converted into heat, which causes an increase in the temperature of the tissue. The rapid heat change leads to thermoelastic expansion of tissue molecules, resulting in the generation of acoustic waves (termed photoacoustic waves or PA waves). The PA waves propagate through the tissue and are detected by ultrasound transducers. The detected acoustic signals are processed using digital signal and image processing algorithms to reconstruct detailed images of the tissue. PAI is often combined with ultrasound imaging to leverage complementary strengths of both techniques. While ultrasound provides high-resolution structural information, PAI adds functional or molecular insights 108.

In PAI, sensitivity is a critical factor that determines the ability of the system to detect weak photoacoustic signals. Sensitivity is quantitatively represented by the noise equivalent pressure (NEP). NEP is the minimum photoacoustic pressure at the imaging target that produces an output signal from the transducer equal to the noise level of the system with a unit of Pa·Hz-1/2. The NEP values of various transducers (piezoelectric transducer: 2 mPA·Hz-1/2, capacitive micromachined ultrasonic transducer [CMUT]: 1.8~2.3 mPA·Hz-1/2, piezoelectric micromachined ultrasonic transducer [PMUT]: 0.84~1.3 mPA·Hz-1/2, optical ultrasound detection: 0.45~486 mPA·Hz-1/2) indicate that micromachined transducers (i.e., CMUTs and PMUTs) are potentially more appropriate transducers for use in PAI applications 109.

PAI uses two primary approaches: photoacoustic computed tomography (PACT) and photoacoustic microscopy (PAM). PACT is optimized for imaging deep tissues, and it can penetrate several centimeters into the tissue. A high-energy pulsed laser is used to illuminate the entire region of interest. Photoacoustic signals generated in deep tissues are detected by transducers with high sensitivity and large directivity. PACT is particularly suitable for whole-body animal studies, making it a valuable tool for preclinical research. To optimize detection, three types of curved-array transducers are used, including arc-shaped, ring-shaped, and hemispherical arc-shaped. Spectroscopic photoacoustic imaging (sPAI) enhances the capability of PAI by utilizing multiple wavelengths of light to extract molecular and functional information about tissues. It is an advanced technique that surpasses structural imaging by analyzing the absorption spectra of different tissue constituents 110. Volumetric photoacoustic imaging (vPAI) utilizes photoacoustic principles to capture images with rich optical contrast. The use of spherical arrays facilitates for rapid 3D scanning with high spatial accuracy, enabling the visualization of deep-seated structures in biological tissues in the NIR spectral window, while further providing superior image quality and rich spectroscopic optical contrast 111.

IVPA imaging is a specialized technique used for IVI. A key component of this system is the IVPA catheter that integrates light delivery and ultrasound detection. An effective IVPA catheter must possess a small dimension, high imaging sensitivity, and sufficient mechanical support to navigate through coronary arteries. Currently, two typical designs are used for IVPA catheters based on the configuration of light delivery and the ultrasound transducer: the co-linear design and the offset design. The co-linear design provides the greatest overlap between the optical and acoustic beams, thereby achieving enhanced imaging sensitivity (1.6 mm diameter prototype 112); however, it poses challenges for miniaturization. The second design, which incorporates an offset (longitudinal or lateral) between the optical and acoustic beams, is commonly preferred in clinical practice due to its significant potential for miniaturization. However, the offset in the catheter may result in signal attenuation when imaging targets are located very near or distant from the transducer 113. To date, the smallest IVPA catheter reported has a diameter of 0.09 mm 114. The key advantages of PAI include its potential for high spatial and temporal resolution, clinically applicable imaging depth, and capability to visualize both endogenous and exogenous chromophores. Common endogenous chromophores include water (both free and bound), oxyhemoglobin (HbO_2_), deoxyhemoglobin (Hb), melanin and lipids. Exogenous agents include small organic molecules such as ICG, inorganic imaging agents, nanoparticles or nanomaterials and nonlinear PA-imaging contrast agents. Exogenous agents can be used as biosensors (for sensing metal ions, pH, enzymes, temperature, hypoxia, ROS, and reactive nitrogen species) by targeting cell membrane molecules or intracellular molecules, and thus provides the clinician with potentially valuable molecular data together with bioimaging 115,116. Many special PA agents are available for CVD imaging 117.

### IVPA imaging application in coronary artery

**Figure 9:**
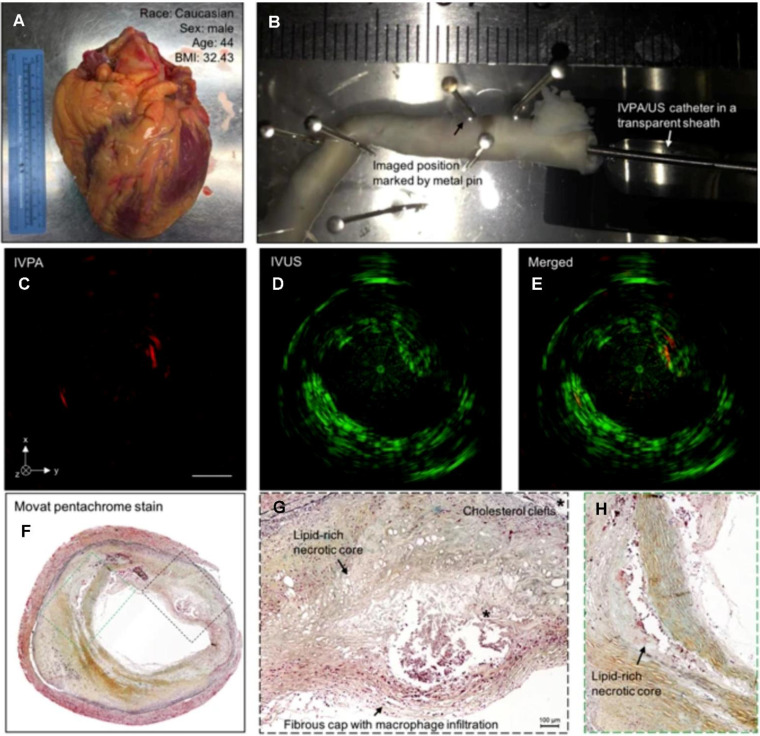
** IVPA-US imaging of human coronary atherosclerosis at 16 fps with comparison to histopathology**. (A) Picture of collected human heart. (B) Scenario picture of *ex vivo* IVPA-US imaging of dissected human coronary artery. The region of interest was marked by metal pin. The catheter and sheath were inserted into the artery lumen. Cross-sectional (C) IVPA, (D) IVUS, and (E) merged images of human coronary artery at the region of interest. (F) Gold-standard histopathology stained with Movat's pentachrome at the region of interest. (G, H) Magnified images of lipid deposition sites corresponding to the dashed boxes in (F). *Indicates the accumulation of cholesterol clefts. Adapted with permission from 120, copyright 2017 The Author(s).

IVPA is a promising modality for identifying and characterizing vulnerable plaques through its ability to image lipid accumulation, intraplaque hemorrhage, and inflammation. Among these, lipid content is the most intensively studied photoacoustic biomarker because of its strong optical absorption in the NIR range. Specific wavelengths between 1180 and 1230 nm are used for lipid imaging via spectroscopic IVPA (sIVPA) 118. sIVPA also utilizes lipid absorption bands at 1.2 and 1.7 μm to differentiate between between plaque lipids and peri-adventitial lipids 119. Lipid-laden plaques were validated through *ex vivo* imaging of atherosclerotic human coronary arteries; the results showed strong correlation with histopathology—the current gold-standard 120,121 (as shown in Figure 9). Exogenous contrast agents can significantly enhance IVPA imaging by improving molecular specificity and sensitivity for detecting key plaques. MMP2 antibody-conjugated gold nanorods (AuNRs-Abs) enabled 3D profiling of protease distribution across plaque strata, revealing remodeling hotspots 122. When combined with IVUS, the hybrid IVPA-IVUS system work synergistically to deliver both molecular and structural information 123. RGDfk-targeted albumin nanoparticles combined with hybrid IVPA-IVUS systems resolved neovessel density and inflammatory burden in rabbit atherosclerosis models 124. IVPA has been validated in *ex vivo* and *in vivo* studies; hence, its clinical translation to guide PCI is a highly promising prospect that could revolutionize the diagnosis and management of atherosclerosis.

### PAI for myocardial structure

PACT has emerged as a complementary imaging modality to conventional cardiac imaging techniques for preclinical research. Various technological improvements have been made to display the normal heart structure. High-frequency PACT pioneered by Park *et al.* achieved unprecedented visualization of intracardiac blood flow, enabling longitudinal studies of functional recovery post-injury 125. *Ex vivo* imaging of excised or perfused murine hearts was performed to study cardiac structure in detail without intracardiac flow dynamics. Lin *et al.* introduced a vPAI system for real-time functional imaging of the isolated Langendorff-perfused heart. This approach achieves centimeter-scale depth penetration and has fast 3D imaging capacity, enabling beat-by-beat visualization of dynamic cardiac activity 126. *In vivo* imaging using vPAI has also been applied to study pulmonary vasculature and heart function under conditions such as chronic hypoxia 127. PACT systems equipped with spherical ultrasound arrays have shown excellent capabilities for real-time imaging of cardiac dynamics, including synchronization between atria and ventricles, as well as perfusion imaging using fluorescence agents such as ICG 128. sPAI using gold nanoparticles has also demonstrated the ability to perform real-time tracking of vascular and cardiac hemodynamics in mice 129.

**Figure 10:**
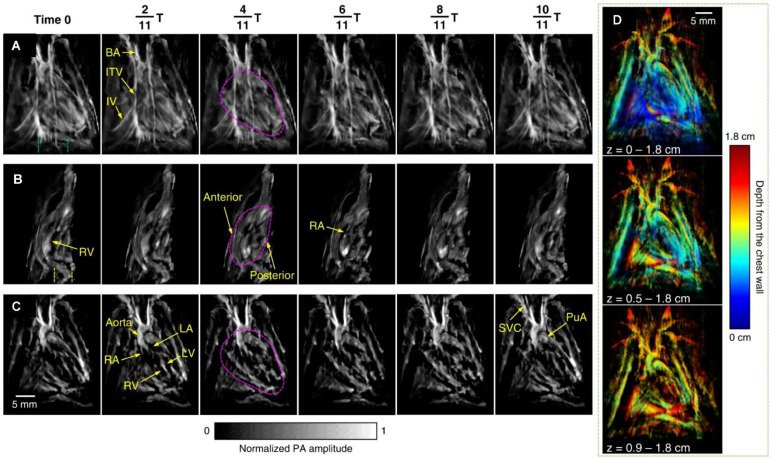
** Rat heart anatomy acquired by the 3D-PACT.** (A) Front view of the heart within a cardiac cycle. The heart is identified by a magenta circle at 411T. (B) Cross-sectional images of the heart on the sagittal plane. Each image is a maximum amplitude projection (MAP) of a slice marked by the green dashed lines in (A). (C) Cross-sectional images of the heart on the coronal plane. Each image is an MAP of a slice marked by the yellow dashed lines in (B). (D) The same data shown with color-encoded depths. Shallower structures were peeled away in the lower images to show the posterior anatomy. Adapted with permission from 134, copyright 2023 The Author(s).

To improve the quality of *in vivo* imaging, Ozsoy *et al.* introduced a sparse PA sensing method for ultrafast 4D imaging of cardiac mechanical wave propagation. This dedicated system is capable of characterizing cardiac mechanical waves in murine models with high contrast, spatial resolution (approximately 115 µm), and sub-millisecond temporal resolution, to provide deeper insights into cardiac function in arrhythmia 130. Mukaddim *et al.* applied spatiotemporal singular value decomposition processing to improve image quality in *in vivo* murine cardiac PAI 131. Incorporating electrocardiogram and respiratory gating allows for precise synchronization with the cardiac cycle, thereby reducing motion artifacts 132. Motion deblurring algorithms further enhance the quality of dynamic cardiac imaging, particularly during rapid cardiac contractions 133. Substantial advancements have been achieved with the application of 3D PACT, which was utilized to reveal distinct cardiac structural and functional changes among healthy, hypertensive, and obese rats. The method identified variations in cardiac chamber size, wall thickness, and hemodynamics. 3D-PACT offers rapid imaging capabilities and nonionizing penetration, enabling comprehensive heart imaging for the diagnosis of animal models 134 (as shown in Figure 10). With continued technological improvements, PACT holds significant promise for clinical translation, particularly in neonatal and pediatric cardiac imaging.

### PAI for myocardium

PAI can enable the direct visualization of myocardial tissues with pathological endogenous components due to ischemia or infarction. Dual-wavelength PAI allows the estimation of blood oxygen saturation, a critical factor in identifying and monitoring myocardial ischemia. The integration of PAI with high-frequency ultrasound provides a non-invasive, real-time imaging approach for studying myocardial ischemia 135. PAI can image MI lesions up to a depth of 10 mm *in vivo*. Quantified results show excellent agreement with the findings of enzyme and histological examinations 136.

**Figure 11:**
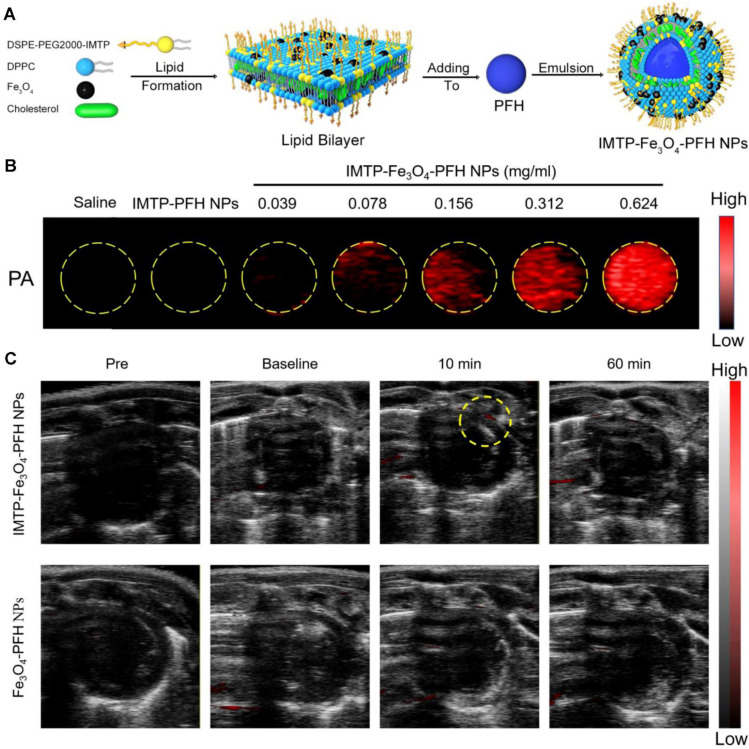
** PA imaging *in vitro* and *in vivo*.** (A) Synthetic procedure of IMTP-Fe_3_O_4_-PFH NPs. (B) PA images of IMTP-Fe_3_O_4_-PFH NPs at different Fe concentrations *in vitro*. (C) *In vivo* PA images of the hearts in model rats after intravenous injection of IMTP-Fe_3_O_4_-PFH NPs or Fe_3_O_4_-PFH NPs pre-operation, baseline, 10 min, and 60 min after injection. Adapted with permission from 141, copyright 2021 The Author(s).

By using an exogenous fluorescent probe, myocardial ischemia injury can be imaged with PAI to display the molecular changes. By analyzing ICG perfusion dynamics at different stages of recovery post-MI, vPAI provides insights into the progression of ischemia and infarction and reveals the effectiveness of therapeutic interventions 137. Lin *et al.* demonstrated that sPAI, combined with ICG, can visualize cardiovascular dynamics on a beat-by-beat basis in murine models of myocardial ischemia 138. Dendritic polyglycerol sulfate-based dPGS-NIR selectively binds P-/L-selectins on activated endothelium, spatially resolving inflammatory hotspots via NIR signal amplification 139. Dendritic polyglycerol sulfate-based dPGS-NIR selectively binds P-/L-selectins on activated endothelium, spatially resolving inflammatory hotspots via NIR signal amplification 140. By integrating ultrasound, photoacoustic, and magnetic resonance capabilities with ischemia-targeting properties, the IMTP-Fe₃O₄-PFH nanoprobe developed by Chen *et al.* provides a comprehensive and non-invasive approach to study myocardial ischemia in preclinical models 141 (as shown in Figure 11). DNA-templated ultrasmall bismuth sulfide (Bi₂S₃) nanoparticles were identified as highly effective PA probes for imaging MI. The *in vivo* imaging results demonstrated a significantly enhanced PA signal in the MI region following intravenous administration of DNA-Bi₂S₃ nanoparticles in the MI/RI model 142. Zhang *et al.* developed fibrin-targeted nanoparticles by co-assembling a fibrin-targeted peptide (CREKA) with ICG to enhance PAI for the non-invasive detection and diagnosis of MI 143. These innovations establish PAI as a transformative modality for decoding ischemic cascades—from oxygen gradients to inflammatory remodeling—with translational potential for personalized cardiac diagnostics.

PAI has been increasingly utilized as an imaging modality to guide and monitor various therapies, including cell therapy and drug delivery. For tracking cell therapy, Berninger *et al.* demonstrated the effectiveness of sPAI and NIRFI as highly sensitive techniques to track intramyocardially injected stem cells labeled with the NIR dye DiR in a rabbit model 144. Complementing this approach, Qin *et al.* engineered semiconducting polymer-based PANPs, demonstrating their utility as high-sensitivity contrast agents for non-invasive human embryonic stem cell-derived cardiomyocytes (hESC-CMs) tracking in cardiac regeneration models 145. For drug delivery optimization, Furdella *et al.* leveraged PAI to resolve stent-eluted therapeutics (DiI-labeled) with submillimeter precision, highlighting its potential to mitigate restenosis through spatially controlled drug release profiling 146. Wang *et al.* further advanced this paradigm with L-arginine-loaded selenium-gold nanocages (AASP), integrating PAI-trackable targeted delivery with multifunctional therapeutic efficacy—reducing apoptosis and fibrosis while restoring myocardial contractility for treating MI/RI 147 (Table 3).

PAI bridges optical molecular specificity and ultrasound depth penetration, offering transformative potential for IHD diagnosis and therapy guidance. PAI offers high spatial-temporal resolution, clinically applicable imaging depth, and the ability to visualize both endogenous chromophores and exogenous agents, enabling molecular and functional insights. It synergizes with ultrasound for structural-molecular imaging and shows promise in detecting vulnerable coronary plaques and myocardial ischemia via oxygen saturation mapping. Preclinical validation highlights its utility in therapy monitoring, such as tracking cell delivery or drug release. However, overcoming penetration limits, refining contrast agents, and accelerating clinical trials are critical to realizing its full potential. Technical challenges include trade-offs in IVPA catheter design and signal attenuation in offset configurations. Dependency on exogenous agents for enhanced specificity raises concerns about clinical translation and safety. While most studies remain preclinical, necessitating validation for clinical adoption.

## Combined application of OI

OI, either independently or in combination with other imaging modalities, is frequently used to evaluate IHD. The development of multimodality intracoronary imaging catheters enhances the assessment of vessel wall pathology and facilitates PCI. Prototypes integrating two or three imaging modalities with complementary attributes have been developed 10,148. OCT has advantages in displaying coronary structure, while NIRFI and PAI show optimal functional display. Therefore, combining these two types of imaging modalities can simultaneously enable structural and functional imaging and achieve complementarity and optimization of evaluation efficiency. Technical feasibility should also be considered while combining these imaging methods, for example, the techniques of OCT, NIRFI and PAI are combined as they require excitation by a laser beam and acquire different optical and photoacoustic signals. Ultrasound imaging plays an equally important role in clinical diagnosis and treatment by virtue of its better structural resolution, accessibility and dynamic imaging capabilities. It closely resembles OI in term of application scenarios and imaging methods and can be combined with various OI imaging techniques 149. In addition to the previously mentioned OCT-NIRFI system, the combination of IVPA-IVUS is most commonly used to assess coronary atherosclerosis, while OCT-IVUS and NIRFI-IVUS systems are also available for this purpose.

An integrated IVUS and IVPA imaging catheter, consisting of a single-element ultrasound transducer and a light delivery system based on a single optical fiber, was developed and applied for *in vivo* imaging of a coronary stent deployed in a rabbit's thoracic aorta under conditions of the luminal blood presence. *In vivo* IVUS/IVPA imaging demonstrates potential for clinical translation 150. Li *et al.* reported that an integrated intracoronary OCT-IVUS system is both feasible and safe for *in vivo* application to detect atherosclerotic plaques in rabbits and swine in an initial demonstration. OCT-IVUS simultaneously provides high resolution and deep penetration capabilities; thus, it is a more robust tool to explore the development of plaques and can facilitate a more precise assessment of vulnerable plaques in patients 151,152. The clinical performance of a novel hybrid OCT-IVUS system was assessed through a multicenter, randomized, non-inferiority trial (PANOVISION); the results demonstrated that this system had good safety and feasibility for use in patients undergoing PCI 153. *In vivo* NIRFI-IVUS imaging was conducted using a bimodal catheter that integrates fluorescence signals induced by ICG into cross-sectional IVUS imaging. *In vivo* ICG accumulation showed correlation with the *ex vivo* fluorescence signal intensity, and the plaques were identified by IVUS 154,155. The obtained results suggest that the NIRFI-IVUS catheter has significant potential to enhance the capabilities of standalone IVUS, facilitate comprehensive vascular disease phenotyping, and improve the assessment and treatment of patients with IHD 156. Multimodal intracoronary imaging systems, such as OCT-NIRFI, IVPA-IVUS, OCT-IVUS and NIRFI-IVUS, offer unparalleled insights into IHD by integrating structural and functional imaging capabilities. These technologies enable comprehensive assessment of vulnerable plaques, improve PCI guidance, and advance clinical management of IHD.

The combination of NIRFI and PA is currently mainly applied in tumor detection. For instance, the novel croconic acid-bisindole dye CR-630, integrated with a morpholine ring, exhibits enhanced lysosome-targeting capability and pH-responsive NIR/PA imaging in breast cancer models, enabling precise tumor visualization 157. Similarly, a self-immolative molecular design utilizing a tunable phenyl ester linker and spirocyclic xanthene fluorophore achieves histone deacetylase-responsive NIRFI/PA imaging and prodrug activation in triple-negative breast tumors. This system combines high-contrast imaging with targeted tumor ablation 158. OI integrated with spectroscopic techniques enhances diagnostic accuracy by merging structural and functional analysis, advancing both research and clinical applications. Notably, the combination of OCT and NIRS has transformed coronary imaging by enabling simultaneous structural and molecular profiling. This multimodal approach improves the identification of high-risk plaques and optimizes stent placement 159,160. Meanwhile, RS provides label-free quantification of tissue biomolecules but is limited by its narrow field of view and lack of morphological context. Integrating RS with OCT in a single platform addresses these limitations, offering comprehensive structural and biochemical tissue characterization for clinical diagnosis 161,162.

### AI-assisted OI

AI is poised to transform medical image analysis, particularly in enhancing the identification and characterization of high-risk atherosclerotic plaques in coronary arteries, thereby advancing IHD management 163. AI tools automate the detection and classification of anomalous coronary artery origins in CT angiography, providing real-time risk alerts and cohort analytics to improve care for rare cardiovascular conditions 164. A CT-derived fractional flow reserve model combining deep learning with level set algorithms surpasses conventional AI platforms in multicenter trials, offering superior stenosis quantification and ischemic lesion detection for automated ischemia assessment 165. In OCT, AI-driven automated analysis enables rapid, precise plaque evaluation, guiding PCI decisions and deepening insights into coronary atherosclerosis pathophysiology 166. Machine and deep learning algorithms significantly enhance OCT image segmentation accuracy, with extensive research validating their efficacy 167. Machine and deep learning algorithms significantly enhance OCT image segmentation accuracy, with extensive research validating their efficacy 168. Deep learning models trained on intravascular OCT data automate coronary artery calcification (CAC) quantification, linking culprit-vessel CAC severity to major adverse cardiovascular and cerebrovascular events 169. As OI becomes more pervasive in clinical practice and generates diverse datasets, AI-assisted technologies will play an increasingly critical role in cardiovascular care.

## Phototherapy for IHD

PDT and PTT are advanced light-based therapeutic approaches capable of eliminating pathological cells with spatiotemporal precision by either generating ROS or increasing temperature. PDT involves the use of three components: photosensitizers, molecules that accumulate in specific cells or organelles and can be activated by light. The photosensitizer selectively localizes within the cell and is capable of targeting multiple cellular organelles, including mitochondria, lysosomes, endoplasmic reticulum, Golgi apparatus, and plasma membranes. Subsequently, the photosensitizer is activated by excitation using light of a specific wavelength. Once activated, photosensitizer interacts with oxygen to generate ROS, which further oxidizes cellular components such as lipids, proteins, and DNA, leading to apoptosis and necrosis. The successful outcome of this process depends on the cell type, the specific photosensitizing agent and its intracellular localization, as well as the intensity and duration of light exposure 170. PTT involves the absorption of light (typically in the NIR range) by nanomaterials, which convert the absorbed energy into localized heat. The resulting hyperthermia selectively damages cells and tissues, leading to cell death. Nanomaterial-mediated PDT and PTT have emerged as promising therapeutic strategies for atherosclerosis and its related diseases 171. PDT or PTT has a direct therapeutic effect, and can also be utilized as an energy conversion tool for stimulating energy to assist other treatment approaches for CVDs.

### PDT for IHD

**Figure 12:**
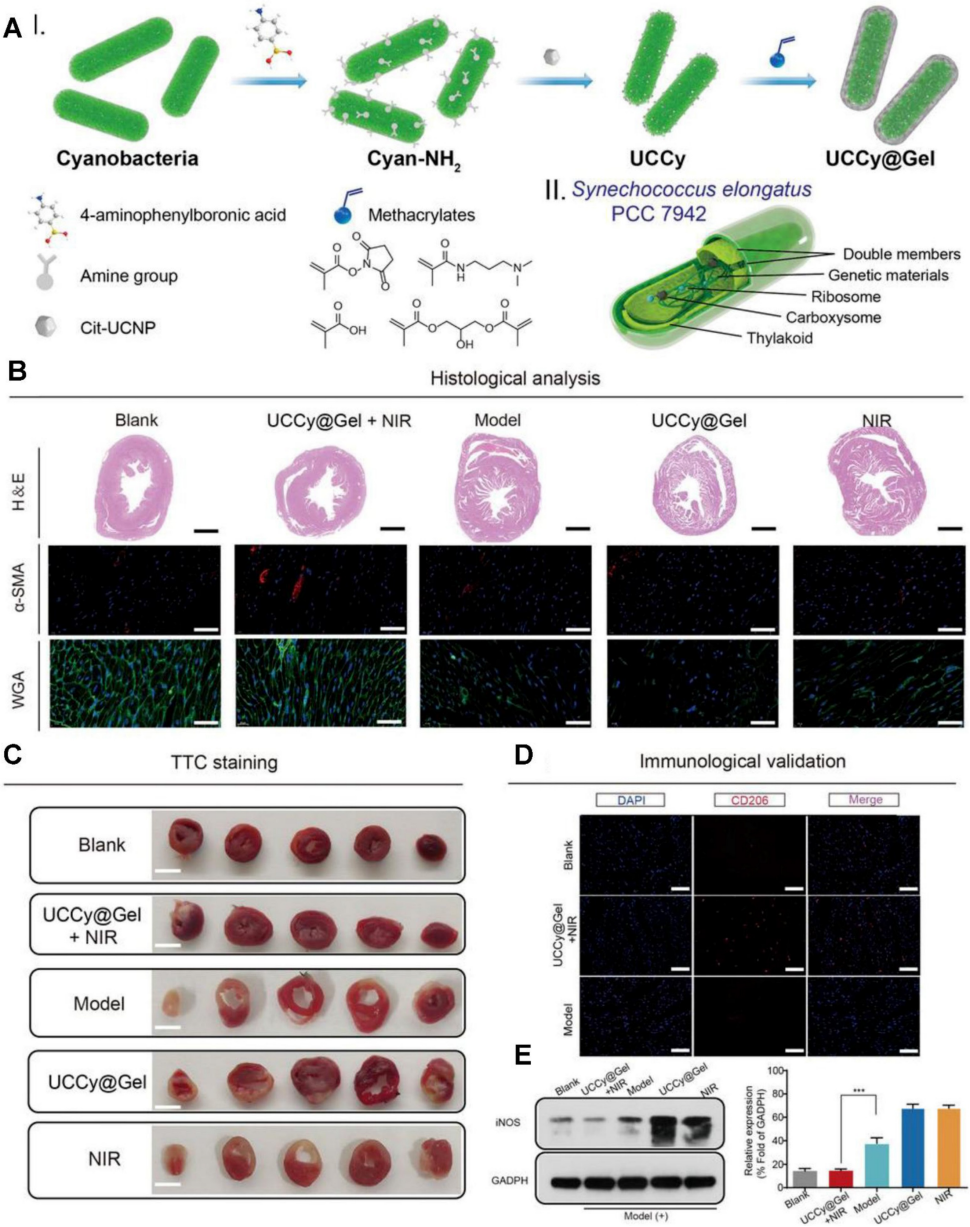
** The cardiac therapeutic function of UCCy@Gel *in vivo*.** (A) I-Schematic illustration for the synthesis of UCCy@Gel, II-the schematic illustration for the structure of cyanobacteria. (B) Heart H&E staining, α-SMA staining, and wheat germ agglutinin staining. (C) CD206 immunofluorescence staining of mice hearts. (D) Western blot and quantitative analysis of iNOS (M1 macrophage marker). Adapted with permission from 175, copyright 2022 The Author(s).

To apply PDT for atherosclerosis, photosensitizers are engineered to selectively accumulate in atherosclerotic plaques. Following the activation of photosensitizers, ROS production leads to the oxidation of lipids, destruction of macrophages, and reduction of plaque volume. Many photosensitizing agents have been developed for the treatment of atherosclerosis or restenosis 127. Jenkins *et al.* investigated the use of intra-arterial, trans-catheter PDT for reducing arterial responses to balloon injury in both coronary and peripheral circulations. 5-Aminolevulinic acid (5-ALA), a precursor of protoporphyrin IX, was used as the photosensitizing agent. PDT significantly reduced the arterial response to injury by inhibiting the proliferation of VSMCs, a major contributor to restenosis 172. PDT is also a promising strategy to prevent neointimal hyperplasia after stent deployment. The use of slotted tube stents in porcine femoral arteries demonstrated the ability of PDT to reduce proliferation of VSMCs and subsequent restenosis risk 173. In balloon-injured arteries of rat and rabbit models treated with PDT, the arterial media remained acellular for weeks to months post-treatment. Intimal hyperplasia did not develop, indicating the long-term efficacy of PDT in suppressing excessive tissue proliferation 174. PDT can also be sued for MI therapy. The UCCy@Gel system developed by Liu *et al.* represents a groundbreaking innovation for MI therapy. By combining photosynthetic oxygen generation with anti-inflammatory effects, this system directly addresses the key pathological mechanisms of MI: hypoxia and inflammation. The minimally invasive nature, requirement of a single injection, and NIR irradiation, make this system a highly promising therapeutic option to prevent and treat MI 175 (As show in Figure 12).

### PTT for IHD

PTT can target lipid-rich, inflamed plaques prone to rupture, stabilize them and reduce the risk of cardiovascular events. To apply PTT as thrombolytic therapy, Dong *et al.* introduced an innovative localized light-Au-hyperthermia (LAH) approach for drug-free blood clot lysis. This technique utilizes gold nanorods (Au NRs) irradiated with NIR light, which generate localized hyperthermia to precisely and rapidly dissolve blood clots. The LAH approach is a minimally invasive alternative for thrombolytic therapy and shows excellent efficacy and biocompatibility in preclinical models 176. To apply PTT as a therapy for myocardial ischemia, Fan *et al.* developed and engineered a vascular endothelial growth factor-loaded, NIR-triggered self-unfolding graphene oxide-poly (vinyl alcohol) microneedle (MN) patch for the treating MI through minimally invasive surgery (MIS). The MN patch effectively promotes neovascularization, reduces myocardial fibrosis, and restores cardiac function, suggesting its potential for clinical application in MIS 177. Feng *et al.* developed a multicomponent hydrogel (EDR@PHCuS HG) that integrates controlled copper-ion release and a mild photothermal effect to synergistically enhance angiogenesis, inhibit cardiomyocyte apoptosis, and repair damaged myocardium following MI. The hydrogel demonstrates significant potential as an advanced therapeutic platform for promoting cardiac function recovery and preventing ventricular remodeling 178.

Phototherapy (PDT/PTT) offers targeted, minimally invasive solutions for IHD, with applications in plaque stabilization, restenosis prevention, and myocardial repair. PDT reduces plaque volume, inhibits restenosis by suppressing vascular smooth muscle cell proliferation, and addresses MI via hypoxia relief and anti-inflammatory effects. PTT stabilizes vulnerable plaques, enables thrombolysis, and promotes cardiac repair through angiogenesis with minimally invasive approaches. However, challenges such as oxygen dependency, light penetration, thermal risks, and translational gaps from animal studies to humans must be addressed for broader clinical adoption.

## Challenges

OI in biological tissues faces a fundamental challenge: achieving high spatial resolution while maintaining sufficient penetration depth. This trade-off arises from photon-tissue interactions, including scattering, absorption, and refractive index heterogeneity. Although intracoronary and intracardiac OI overcome limitations related to tissue penetration, they require invasive procedures, which may not be suitable for all patients. Invasiveness increases procedural risks and limits its routine application in broader patient populations. Cardiovascular tissues, such as the heart and coronary artery, are in constant motion from heartbeat and respiration; this complicates real-time imaging and requires advanced signal processing. The lack of standardization in OI systems and techniques complicates their integration into routine clinical workflow. Addressing the impact of optical properties on imaging stability and ensuring high imaging quality are critical challenges that require urgent attention.

Endogenous molecules such as hemoglobin and melanin have inherent optical and acoustic properties but lack the specificity and signal amplification required for precise lesion detection in IHD. The ideal fluorescent or photoacoustic probe should exhibit long fluorescence emission time, high photoacoustic signal intensity, strong photostability and excellent aqueous solubility, minimal or no toxicity, favorable metabolic properties, high quantum yield, and lesion-specific enrichment. To date, only three NIR fluorescent probes have been approved by the FDA for clinical use: ICG, MB and 5-ALA. Hence, further development of advanced targeted fluorescent or photoacoustic probes is required. Nanocarrier systems co-encapsulate contrast agents, enhancing sensitivity via plasmonic resonance or metal-enhanced fluorescence, while antibody-/peptide-functionalized variants achieve subcellular targeting. However, long-term retention and degradation of nanomaterials may result in toxic effects, such as inflammation or organ damage; moreover, slow clearance of nanomaterials from the body hinders clinical safety, which remains the largest obstacle to their clinical transformation. Additionally, most studies on optical probes are currently at the basic or preclinical stage, with limited human data to demonstrate the efficacy and safety of these probes in IHD. A considerable amount of work is pending for the successful clinical translation of OI.

Compared to conventional treatments for IHD, optical therapy is still in its early stages of development; this limits its clinical adoption and effectiveness. This technology needs further advancement to meet the standards of established treatment methods. Translating optical therapy from the laboratory or experimental setting to practical, real-world clinical application for IHD treatment remains a considerable challenge. This involves overcoming both technical barriers and regulatory hurdles. Current research lacks sufficient data on the long-term safety of optical therapy, particularly regarding potential toxic effects, with specific gaps in understanding systemic toxicity from residual photosensitizers post-treatment and strategies to mitigate heat damage from prolonged light exposure. Additionally, there are no well-established methods to monitor patient prognosis over time after optical therapy. A major obstacle is ensuring that optical therapy can provide treatment efficacy equivalent to, or better than, surgical interventions. In conclusion, although optical therapies have a promising potential because of their non-invasive nature, significant efforts are required to address their safety, efficacy, and cost issues before they can be used as mainstream alternatives for treating IHD.

The clinical translation of optical theranostics hinges mainly on three priorities: bridging interdisciplinary talent gaps, reducing costs through technological innovation, and generating robust clinical evidence. Endovascular interventions demand specialized skills in imaging-guided device navigation and time-sensitive clinical judgment, while surgical integration of OI requires seamless engineering-clinical teamwork. Cost barriers primarily arise from high-end imaging infrastructure and rigorous production standards for nanotheranostic agents such as gold nanoparticles. Clinical validation remains constrained, with minimal agents progressing to Phase III trials. Current progress in nanotechnology, imaging modalities, and cross-disciplinary collaboration models supports expanded implementation, contingent on sustained research funding and structured clinician-engineer partnerships.

## Further perspectives

Emerging technological paradigms in NIR-II optical coherence tomography have redefined depth-resolved vascular interrogation, overcoming historical limitations of visible/NIR-I optical penetration constraints. A spectral domain ultrahigh resolution OCT system operating in a NIR-II window range using a supercontinuum light source was constructed; the system achieved an axial resolution of 2.6 µm in air with deeper penetration into tissues than that achieved with visible or NIR-I light 179. Gold superclusters (AuSCs) were developed and imaged with the OCT NIR-II laser, which enabled the detection of vascular inflammation^180^. Song *et al.* developed a polymer nanoplatform integrating NIR-II PA for precise thrombus detection and a dual-mode antithrombotic mechanism combining localized photothermal therapy with on-demand nitric oxide release, achieved through a fibrin-targeted semiconducting polymer design, demonstrating superior thrombus resolution and blood flow restoration in multiple vascular models 181. The superior acquisition speed of NIR-II imaging and the efficient selective targeting of NIR-emitting nanoparticles facilitated rapid *in vivo* imaging of the infarcted heart within a few minutes after the occurrence of the acute infarction event 92. These advancements underpin volumetric optical angioarchitectonics, now enabling 3D hemodynamic profiling for atherosclerotic vulnerability stratification 48. Athough endogenous fluorescent molecules can be used for imaging, molecule-specific imaging has high application prospects. The development of organic molecular probes is conducive to clinical translation and could certainly facilitate the diagnosis and treatment of IHD. The development of exogenous fluorescent molecules can also contribute to the clinical transformation of phototherapy. In addition to NIRFI and PAI, molecular contrast OCT techniques could be promising alternatives and provide enhancements in tissue imaging, for example, pump-probe OCT, pump suppression OCT, magnetomotive OCT, and photothermal OCT 182. Palma-Chavez *et al.* engineered VCAM-1-functionalized PLGA microparticles encapsulating MB, demonstrating dual-modal pump-probe/photothermal OCT detection of inflammatory endothelium in atherosclerosis 183. Parallel innovations include photothermal OCT identification of macrophage-rich plaques via gold-iron oxide nanoclusters (nanorose) in rabbit models 184. and catheter-based magnetomotive OCT using RGD-conjugated magnetic microspheres to map αvβ3 integrin overexpression in atherosclerotic aortas 185. The combination of nanoparticle-based targeting with OCT is a robust approach for non-invasive, real-time monitoring of atherosclerosis and other vascular diseases.

The integration of intelligent nanotechnology enhances PDT's precision and therapeutic efficacy through multifunctional nanoplatforms that demonstrate synergistic effects in targeted therapy, potentially reducing photosensitizer-induced systemic toxicity 186. Zou *et al.* reported CD68 antibody-conjugated Ce6-loaded liposomes that enhance atherosclerosis-targeted PDT through laser-activated ROS generation, simultaneously promoting foam cell autophagy, inhibiting vascular smooth muscle migration, and stabilizing atherosclerotic plaques 187. Liu *et al.* engineered light-sensitive polymer nanocarriers integrated with wireless dual-wavelength microLED devices, which overcome deep-tumor PDT limitations through programmed photosensitizer activation via sequential light irradiation, achieving enhanced therapeutic efficacy with reduced drug doses in preclinical studies 188. Beyond traditional PDT and PTT, emerging light-responsive platforms show particular promise for IHD treatment. Huang *et al*.'s redox-responsive TPE-ss covalent organic framework (COF) nanocarriers effectively mitigated MI/RI by reducing cardiomyocyte apoptosis while improving cardiac function and survival rates, establishing COFs as promising smart carriers for peri-reperfusion therapy in IHD 189. These advancements highlight nanotechnology's transformative potential in developing precision phototherapeutics. Future directions should focus on creating multifunctional platforms that integrate real-time monitoring, adaptive drug release, and combinatorial therapeutic approaches to address complex cardiovascular pathologies.

Hybrid imaging, which involves the combination of structural imaging and molecular imaging through multimodal imaging techniques, is also the direction of future development in the field of OI imaging. Beyond the combination of different OI imaging techniques, the combination of OI and ultrasound can provide advantages over existing established cardiac imaging technology and emerging technologies to guide the precise diagnosis and treatment of CVDs in clinical settings. The combination of other imaging modalities such as CT, MRI and nuclear medicine, with optical therapy should also be considered. AI enhances hybrid imaging by optimizing image reconstruction and automating biomarker quantification. Among the imaging modalities, PET/SPECT and NIR imaging provide synergistic properties that result in deep tissue penetration and up to cell-level resolution. Dual-modal PET/SPECT-NIR agents are commonly combined with biomarkers to engage biomolecules overexpressed in tissue, thereby enabling selective multimodal visualization of the lesions 190. A novel bimodal contrast agentwith high liver specificity enables early-stage hepatocellular carcinoma detection through sensitive CT/NIR imaging, demonstrating noninvasive tumor-targeting efficacy and vascular visualization in multiple preclinical models 191. Future advancements should focus on optimizing multimodal combinations for IHD management.

Optical platforms have gained prominence in bioengineering for developing wearable biosensors that enable non-invasive monitoring 192-194. Recent advances overcome limitations of conventional chemiluminescent-based systems; for instance, a novel biosensor demonstrated rapid MI diagnosis by measuring cardiac troponin concentrations with miniaturization potential 185. Breakthroughs in sensitivity include Niu *et al.*'s fiber-integrated optofluidic immunosensor, which employs whispering gallery mode probes and microwave photonic analysis to achieve ultrahigh-resolution detection of cardiac troponin I-C complexes—surpassing existing fiber biosensors by 10²-10⁴-fold in resolving power 195. Further integration with wearable technology is exemplified by a machine learning-enhanced wrist-worn infrared spectrophotometric sensor, which noninvasively predicts elevated troponin-I levels in acute coronary syndrome patients, correlating with cardiac dysfunction and coronary stenosis 196. Future research may focus on integrating multi-modal sensing technologies, refining machine learning algorithms for real-time data interpretation, and scaling fabrication for widespread clinical adoption.

Augmented reality (AR) systems are demonstrating transformative potential in surgical NIRFI guidance. An intraoperative AR goggle system achieves real-time tumor margin visualization in animal models through dichroic beam splitter-mediated integration of NIRFI with visible-light anatomy, maintaining spatial consistency between fluorescence and anatomical views without algorithmic compensation despite device distortion 197. Separately, a coaxial infrared AR system successfully mapped myocardial ischemia dynamics during porcine cardiac surgery, revealing characteristic temperature gradients and concentric penumbra patterns during coronary occlusion, suggesting utility for intraoperative perfusion monitoring^198^. These AR platforms exemplify the convergence of optical engineering and surgical practice, offering real-time tissue characterization with submillimeter spatial registration. Future developments should focus on clinical validation in human trials, miniaturization of optical components for ergonomic use, and integration with multimodal imaging to create comprehensive surgical navigation ecosystems.

## Conclusion

Optical theranostics could serve as a foundation for precision medicine-based diagnosis and treatment of IHD. By providing high-resolution, non-invasive imaging modalities and enabling light-based therapies, OI platforms can offer more personalized, minimally invasive interventions for patients with IHD. However, challenges such as tissue penetration, motion artifacts, and clinical validation must be addressed before these technologies are fully integrated into routine clinical practice. The ongoing advancements in nanotechnology, contrast agents, and multimodal imaging systems will play a critical role in overcoming these challenges and releasing the full potential of optical theranostics in cardiovascular care.

## Figures and Tables

**Table 1:** Fluorophores and molecular targets for diagnosing coronary atherosclerosis.

Fluorophores	Types	Targets	Artery change	Imaging type	Refs.
ICG	Small molecule	Macrophages and lipid	Inflammation	NIRFI-OCT	55 56
scFv-Fc-2c antibody-Alexa Fluor 647	Antibody	Galectin-3	Inflammation	NIRFI	57
Anti-ICAM-1 SDA/CBHP- IR800CW/IR660CW	Antibody/Peptide	ICAM-1/ unpolymerized type I collagen	Inflammation	NIRF-IVUS	58
LO1-750 (VivoTag-S 750)	Antibody	Ox-LDL	Atheroma lipid-rich necrotic core	NIRFI	59
LO9-750 (VivoTag-S 750)	Antibody	Ox-LDL	Atheroma lipid-rich necrotic core	NIRFI	60
FTP11-Cy7/AlexaFluor 750/ ICG	Peptide	Macrophages /Fibrin deposition	Inflammation	NIRFI-IVUS	61
FTP11-CyAm7	Peptide	Fibrin deposition	Prothrombotic stent	NIRFI-OCT	62
CLIO-CyAm7	Nanoparticles	Macrophages	Inflammation	NIRFI	63
IR-QDs	Nanoparticles	Macrophages	Inflammation	NIRFI-OCT	64
MMR-Lobe-Cy	Nanodrug	Macrophage mannose receptor	Inflammation	NIRFI-OCT	65
Fe_3_O_4_-Cy7	Nanoparticles	Macrophages	Inflammation	NIRFI-MRI	66
PLGA-cRGD-PFH-ICG	Nanoparticles	Microthrombi	Activated platelets	NIRFI-IVPA	72

**Table 2:** Fluorophores and molecular targets for NIRFI of the myocardium.

Fluorophores	Type	Targets	Myocardial change	Imaging type	Refs
F16(s)	Small molecule	Mitochondria	Myocardial perfusion imaging	NIRFI	79
TPABTBP	Small molecule	Lipid droplet in cardiomyocyte ferroptosis	MI/RI	NIRFI	80
ApoPep-1-FITC/Flamma774	Peptide	Myocardial cell apoptosis and necrosis	Acute MI	NIRFI	81
Cy5.5-annexin V	Protein	Phosphatidylserine	MI/RI	NIRFI	82
Dextran-TO	Nanoprobe	Extracellular nucleic acids	Myocardial ischemia injury	NIRFI	83
AND-Cy5.5	Nanoparticles	Autophagosomes of cardiomyocyte autophagy	Myocardial injury	NIRFI /MRI	84
SCIO-ICG-CRT-CPPs NPs	Nanoparticles	Transferrin receptor 1	MI/RI	NIRFI /MPI/MRI	85
ICG	Small molecule	Mesenchyme	MI/RI	NIRFI	86
Cy5.5-MMP2/9 targeted peptide	Peptide	Mesenchyme	MI	NIRFI	87
BBEB	Small molecule	Peroxynitrite (ONOO⁻)	Acute MI	NIRFI	88
NOF5/Cy3-SCLMs	Nanoprobe	Peroxynitrite (ONOO⁻)	MI/RI	NIRFI	89
CLP NPs	Nanoparticles	ROS	MI/RI	NIRFI	90
Pep/BDP-NO_2_@Lip	Nanoprobe	Angiotensin II receptor 1	Myocardial hypoxia	NIRFI	91
AngII-Ag_2_S NDs	Nanodots	Angiotensin II receptor 1	MI	NIRFI -II	92
MnO-PEG-Cy5.5	Nanoparticles	Mesenchyme	MI	NIRFI /MRI	93
IR783-R-SPION-CREKA	Nanoparticles	Fibrin	Microthrombosis in MI/RI	NIRFI /MRI	94
CLIO-Cy5.5	Nanoparticles	Macrophage	MI	NIRFI /MRI	95
T-MBs-ICG	Microbubbles	Fibrin	CMD	NIRFI /US	96
IMTP- MBs-ICG	Microbubbles	Ischemic myocardium	CMD	NIRFI /US	97
OPN@PFP-DiR NPs	Nanoparticles	Cardiac fibrosis	Cardiac fibrosis	NIRFI /US	98
DiD	Small molecule	MSCs tracking	MI therapy	NIRFI	100
IR-786	Small molecule	MPCs tracking	MI therapy	NIRFI	101
Alexa Fluor 790 /Alexa Fluor 647	Small molecule	CPC-EVs tracking	MI therapy	NIRFI	102
PMSN-siRNA-PEI	Nanoparticles	SiRNA delivery in mesenchyme	MI therapy	NIRFI	103
Dil-CD47 Evs	Small molecule	MiR-21a loaded in EVs	MI/RI therapy	NIRFI	104
Angiotensin 1-CdSe/ZnS QDs	Nanoparticles	Ischemic myocardium	MI/RI therapy with cystathionine-γ-lyase	NIRFI	105
HI@PSeP-IMTP NPs	Nanoparticles	Ischemic myocardium	MI/RI therapy with hesperadin	NIRFI /PAI	106
AS-I/S NCs	Nanostructures	Ischemic myocardium	MI/RI therapy with antioxidant agents	NIRFI /PAI	107

**Table 3:** Fluorophores and molecular targets for PAI of the myocardium.

Fluorophores	Type	Targets	Myocardial change	Imaging type	Refs
ICG	Small molecule	Myocardial perfusion	Myocardial ischemia/MI	vPAI/ sPAI	**137,138**
dPGS-NIR	Small molecule	P- and L-selectins	MI	PAI	**139**
CNA35-GP@NPs	Nanoparticles	Collagen fiber	Myocardial fibrosis	PAI/US/CT	**140**
IMTP-Fe_3_O_4_-PFH NPs	Nanoparticles	Ischemic myocardium	Myocardial ischemia	PAI/US/MRI	**141**
DNA-Bi_2_S_3_ NPs	Nanoparticles	Mesenchyme	MI	PAI	**142**
CREKA-ICG-LIP NPs	Nanoparticles	Fibrin	MI	PAI	**143**
DiR	Small molecule	MSCs tracking	MI	sPAI/NIRFI	**144**
SPs-PANPs	Nanoparticles	hESC-CMs	MI	PAI	**145**
DiI	Small molecule	Myocardial perfusion/drug-eluting stent	Drug delivery tracking	PAI	**146**
AASP	Nanocages	Cardiomyocytes	MI/RI therapy	PAI	**147**

## Abbreviations

IHD: ischemic heart disease
CVDs: cardiovascular diseases
CAS: atherosclerosis-driven stenosis
SPECT: single-photon emission computed tomography
MRI: magnetic resonance imaging
PET: positron emission tomography
OI: optical imaging
OCT: optical coherence tomography
NIRFI: near-infrared fluorescence imaging
PAI: photoacoustic imaging
NIRS: near-infrared spectroscopy
RS: Raman spectroscopy
ROS: reactive oxygen species
MI: myocardial infarction
IVI: intravascular imaging
NO: nitric oxide
LDL: low-density lipoprotein
Ox-LDL: oxidized low-density lipoprotein
VCAM-1: vascular cell adhesion molecule 1
ICAM-1: intercellular adhesion molecule 1
VSMCs: vascular smooth muscle cells
ECM: extracellular matrix
MMPs: matrix metalloproteinases
RBC: red blood cells
Atg: autophagy-related gene protein
DAMPs: danger-associated molecular patterns
GPX: glutathione peroxidase
GSH: glutathione
GSSG: glutathione disulfate
LC3: microtubule-associated protein light chain 3
MLKL: mixed-lineage kinase domain-like kinase
RIPK: receptor-interacting protein kinase
TLR: toll-like receptor
TNFR: tumor necrosis factor receptor
STED: Stimulated Emission Depletion Microscopy
PALM: Photoactivated Localization Microscopy
STORM: Stochastic Optical Reconstruction Microscopy
MI/RI: myocardial ischemia/reperfusion injury
IVUS: intravascular ultrasound
PCI: percutaneous coronary intervention
TD-OCT: Time Domain OCT
FD-OCT: Frequency Domain OCT
TCFA: thin cap fibroatheroma
AI: artificial intelligence
TEM: transmission electron microscopy
NIRAF: NIR autofluorescence
CCD: charge-coupled device
ICG: indocyanine green
2D: two-dimensional
NIR-II: second near-infrared window
3D: three-dimensional
PPAR: peroxisome proliferator-activated receptor
CEA: carotid endarterectomy
SDA: single-domain antibody
CBHP: collagen-binding hairpin peptide
IVPA: intravascular PA
MB: methylene blue
LD: lipid droplet
MPI: magnetic particle imaging
CMD: coronary microvascular dysfunction
IMTP: ischemic myocardium-targeting peptide
MSCs: mesenchymal stem cells
MPCs: multipotent progenitor cells
CPC-Evs: cardiac progenitor cell-derived extracellular vesicles
NEP: noise equivalent pressure
CMUT: capacitive micromachined ultrasonic transducer
PMUT: piezoelectric micromachined ultrasonic transducer
PACT: photoacoustic computed tomography
PAM: photoacoustic microscopy
sPAI: spectroscopic photoacoustic imaging
vPAI: volumetric photoacoustic imaging
HbO2: oxyhemoglobin
Hb: deoxyhemoglobin
sIVPA: spectroscopic IVPA
MAP: maximum amplitude projection
CAC: coronary artery calcification
hESC-CMs: human embryonic stem cell-derived cardiomyocytes
5-ALA: 5-Aminolevulinic acid
LAH: light-Au-hyperthermia
MN: microneedle
MIS: minimally invasive surgery
COF: organic framework
AR: augmented reality
